# Osteostimulatory effect of biocomposite scaffold containing phytomolecule diosmin by Integrin/FAK/ERK signaling pathway in mouse mesenchymal stem cells

**DOI:** 10.1038/s41598-019-48429-1

**Published:** 2019-08-15

**Authors:** S. Viji Chandran, M. Vairamani, N. Selvamurugan

**Affiliations:** Department of Biotechnology, School of Bioengineering, SRM Institute of Science and Technology, Kattankulathur, Tamil Nadu India

**Keywords:** Nanoparticles, Tissue engineering

## Abstract

Non-availability of an ideal alternative for autografts in treating critical-size bone defects is a major challenge in orthopedics. Phytocompounds have been proven to enhance osteogenesis via various osteogenic signaling pathways, but its decreased bioavailability and increased renal clearance limit its application. In this study, we designed a biocomposite scaffold comprising gelatin (Gel) and nanohydroxyapatite (nHAp) incorporated with diosmin (DM) and we investigated its bone forming potential *in vitro* and *in vivo*. Physiochemical characterization of the scaffold showed that DM had no effect on altering the material characteristics of the scaffold. The addition of DM enhanced the osteoblast differentiation potential of the scaffold in mouse mesenchymal stem cells at both cellular and molecular levels, possibly via the integrin-mediated activation of FAK and ERK signaling components. Using the rat tibial bone defective model, we identified the effect of DM in Gel/nHAp scaffold on enhancing bone formation *in vivo*. Based on our results, we suggest that Gel/nHAp/DM can be a potential therapeutic agent in scaffold-mediated bone regeneration.

## Introduction

Bone and cartilage ailments, such as osteoporosis, arthritis, and spinal injuries, etc., although not life threatening, diminish the quality of life with continuous pain, discomfort and restricted locomotion^1^. The conventional treatments of tissue graft and artificial prosthetics fail to render a permanent solution owing to high cost, repetitive surgery, and donor site morbidity^2,3^. Bone tissue regeneration via bioactive scaffold can ameliorate the quality of life due to its advantageous bone regenerative potential^4^. Phytocompounds are known to have several medicinal properties and incorporation of phytocompounds into scaffold matrices is advantageous for enhanced bone regeneration^5–7^. It can also be considered a possible alternative for growth factor (GF)-mediated therapy.

The major drawback of scaffold-mediated regenerative therapies is the failure in mimicking the extracellular matrix (ECM) environment for cell adhesion. Surface modification strategies, such as coating the scaffold surface with several integrin-specific ligands, like RGD, GFOGER, are currently being investigated for enhanced cell adhesion^8,9^. These techniques were highly successful in cell culture models, but failed to reproduce the same results *in vivo*^8^. This was possibly due to the synthetic peptide interactions with other body fluid proteins upon implantation^8^. Even though gelatin (Gel) contains RGD binding peptides, the lack of availability of the same for integrin binding due to a cross-linking reaction with other compounds in the composite might be a disadvantage^10^. These RGD binding peptides in Gel may be protected to a greater extent by dissolving it in acetone:water solution containing 1-ethyl-3-(3 dimethylaminopropyl) carbodiimide hydrochloride (EDC)^11^. Compounds like ascorbic acid, which can enhance deposition of integrin binding proteins or peptides, such as collagen, on to the implant surface from the surrounding cells upon implantation, can be of higher clinical relevance^12,13^.

It has been suggested that many of the phytocompounds with an anti-diabetic property may possess osteo-inductive potential^14–16^. A limited knowledge of the mechanism of action of phytocompounds and their low bioavailability limit the full range application of phytocompounds in tissue regeneration^17^. Diosmin (DM), a phytomolecule, is an ironside flavonoid glycoside mainly present in citrus fruits. It has been studied for its anti-diabetic, anti-oxidant, and anti-inflammatory properties^18–20^. Daflon®, a commercially available drug containing 90% DM and 10% hesperidin, is used for the treatment of chronic venous disease by inhibiting prostaglandin synthesis and free radical scavenging^21,22^. To our knowledge, no report is available about the role of DM in promoting osteogenesis or in any other tissue engineering applications. The water-insoluble nature of DM is a major limitation for affecting its bioavailability and increases its renal clearance. Several studies showed that combining such water-insoluble compounds with natural hydrophilic polymers might alter the nature of the compound providing sustained and prolonged delivery of compounds and better absorption to the target site^23–26^.

Collagen (Col) and hydroxyapatite (HAp) are two main components of bone^3,12,27^. Gel is a partially hydrolyzed form of Col having the RGD binding peptides for successful cell attachment during implantation. It is biocompatible in nature, with low immunogenic reactions, and also provides ease of handling^23,28^. HAp is a carbonated apatite that can regulate bone metabolism via the release of calcium and phosphate ions. Generally, calcium ions influence osteoblast proliferation and osteoclast regulation, while phosphate ions regulate osteoblast apoptosis and mineralization rate^29,30^. In this study, we aimed to enhance the bioavailability of DM by incorporating it into a Gel/nHAp scaffold, and to study its osteogenic potential *in vitro* along with an integrin-mediated cell signaling cascade, and finally, to identify its potential ability towards bone forming using an *in vivo* rat tibial bone defective model.

## Materials and Methods

### Materials

Diosmin (DM; MW: 608.54 g/mol, purity: ≥90%), nanohydroxyapatite (nHAp, <200 nm particle size), 1-ethyl-3-(3 dimethylaminopropyl) carbodiimide hydrochloride (EDC), fluorescein diacetate (FDA) and 3-(4,5-Dimethylthiazol-2-yl) 2,5-Diphenyltetrazolium Bromide (MTT) were obtained from Sigma Aldrich (St. Louis, MO, USA). C3H10T1/2 cells (mouse mesenchymal stem cells; mMSCs) were obtained from National Centre for Cell Science (NCCS), Pune, India. Dulbecco’s Modified Eagle’s Medium (DMEM) was obtained from Lonza-BioWhittaker (Walkersville, MD, USA) and Fetal Bovine Serum (FBS) was purchased from Invitrogen^M^ (Waltham, MA, USA). 5-bromo-4-chloro-3′-indolyl-phosphate-nitro-blue tetrazolium (BCIP-NBT) was procured from Amresco® (OH, USA). Gelatin (Gel) and the antibodies for western blot analysis were obtained from Santa Cruz Biotechnology (Dallas, TX, USA). Westar Supernova-chemiluminescent substrate was purchased from Cyanagen (Bologna, Italy). Phosphate buffered saline (PBS) was procured from HiMedia Laboratories Pvt. Ltd. (Mumbai, India). Acetic acid, ethanol, and other reagents used were of analytical grade.

## Methods

### Preparation and characterization of Gel/nHAp and Gel/nHAp/DM scaffolds

The Gel/nHAp/DM scaffold was prepared using the lyophilization (freeze-drying) technique. Briefly, 1% Gel was dissolved in water and DM dissolved in DMSO was added dropwise to the solution making a final concentration of DM in the solution to be 10–100 μM. After stirring for an hour, 1% nHAp was added to the solution and stirred for 5 h to obtain a uniform suspension and casted into plates. Overnight freezing of plates at −20 °C was performed followed by lyophilization at −40 °C and 0.09 mbar for 48 h. After lyophilization, the scaffold was incubated in 50 mM EDC [acetone: water (8:2 v/v)] solution for cross-linking at 4 °C, and then lyophilized. The obtained scaffold was subjected to physicochemical and material characterization using Scanning electron microscope (SEM), Fourier-transform infrared spectroscopy (FTIR), X-ray diffraction (XRD), swelling, protein adsorption, degradation, and biomineralization studies according to previously described protocols^31,32^.

Briefly, in swelling studies, the scaffolds were incubated in 1X PBS solution for varying periods, and the swelling ratio was calculated as follows:

$${\rm{Swelling}}\,{\rm{ratio}}=[{\rm{Final}}\,{\rm{weight}}-{\rm{Initial}}\,{\rm{weight}}]/{\rm{Initial}}\,{\rm{weight}}$$

Protein adsorption study was carried out by incubating the scaffolds in 1% FBS containing medium for varying periods, and the amount of proteins adsorbed was determined by the indirect method of protein estimation using Bradford assay as follows:

$$\begin{array}{rcl}{\rm{Amount}}\,{\rm{of}}\,{\rm{proteins}}\,{\rm{adsorbed}} & = & {\rm{Initial}}\,{\rm{amount}}\,{\rm{of}}\,{\rm{proteins}}\,{\rm{incubated}}\\  &  & -{\rm{Final}}\,{\rm{amount}}\,{\rm{of}}\,{\rm{unadsorbed}}\,{\rm{proteins}}\,{\rm{in}}\,{\rm{incubated}}\,{\rm{solution}}\,({\rm{supernatant}})\end{array}$$

Degradation study was performed by incubating the equally weighed scaffolds in 1X PBS solution containing 10,000 U/L lysozyme for varying time periods, and the percentage degradation rate was calculated as follows:

$$ \% \,{\rm{Degradation}}=[({\rm{Initial}}\,{\rm{weight}}-{\rm{Final}}\,{\rm{weight}})/{\rm{Initial}}\,{\rm{weight}}]\ast {\rm{100}}$$

### Drug release studies

Equally weighed scaffolds were incubated in 1X PBS at 37 °C in an orbital shaker at 50 rpm. Then, 200 μl of samples was collected at different time intervals and the absorbance of DM released from the scaffold was read at 268 nm^33^. The concentration of DM was calculated using the slope of the DM standard graph. HPLC analysis was performed for qualitative analysis of DM released from scaffold and compared with standard. Methanol:water in a ratio of 70:30 was used as the mobile phase for the analysis.

### Cyto-compatibility of scaffolds

C3H10T1/2 cells were cultured in DMEM containing 10% FBS and 1X pen/strep amphotericin B solution. The scaffold was ethanol sterilized for 30 min followed by equilibration with 1X PBS overnight and then incubated with 10% FBS containing DMEM for 1 h and used for direct cell seeding. Cells (1.5 × 10^5^ cells/well) were seeded onto the scaffold and cultured in DMEM containing 10% FBS for 72 h. Scaffold in the size of 20 mm diameter and 5 mm thickness was used in cell culture experiments. Following the incubation, the cell-seeded scaffold was incubated with MTT solution for 2 h. Formazan crystals were then dissolved in DMSO and quantified at 570 nm. Cell morphology assessment was performed using FDA staining. Cells were seeded on to scaffold and incubated for 72 h in DMEM containing 10% FBS. The cell-seeded scaffold was washed in 1X PBS and incubated with FDA solution for 20 min in dark. Visualization was carried out using a fluorescent microscope (450–490 nm)^34^.

### ALP and von Kossa staining

C3H10T1/2 cells were seeded onto a scaffold and cultured for 7 and 14 days in DMEM containing 10% FBS for ALP and von Kossa staining, respectively. Following the treatment period, the cell-seeded scaffold was fixed with 10% formalin solution for 15 min. ALP staining and von Kossa staining were performed according to the previously described protocol^23^.

### Reverse transcriptase real-time or quantitative (RT-qPCR) analysis

C3H10T1/2 cells were seeded on the sterilized scaffold and cultured for 7 and 14 days. Total RNA isolation was performed using the TRIzol method and followed by cDNA synthesis using the iScript cDNA synthesis kit (Bio-Rad, Hercules, CA, USA) according to the manufacturer’s protocol. qPCR analysis was carried out using SYBR green (Takara, Kyoto, Japan) in QuantStudio 3 v1.4. Relative mRNA expression was calculated using the ΔΔCt method of quantification. The primers used in this study are shown in Table 1. The primer sequences of integrins were obtained from MGH primer bank (https://pga.mgh.harvard.edu/primerbank/).

**Table 1:** A list of primer sequences used in qPCR.

Genes	5′-3′ Sequences	Reference
Runx2	Forward- CGCCTCACAAACAACCACAG Reverse- TCACTGTGCTGAAGAGGCTG	^7^
ALP	Forward- TTGTGCCAGAGAAAGAGAGAGA Reverse- GTTTCAGGGCATTTTTCAAGGT	^7^
Col-1	Forward- TAACCCCCTCCCCAGCCACAAA Reverse- TTCCTCTTGGCCGTGCGTCA	^7^
OC	Forward- ATGGCTTGAAGACCGCCTAC Reverse- AGGGCAGAGAGAGAGGACAG	^7^
Osterix	Forward –ACTGGCTAGGTGGTGGTCAG Reverse –GGTAGGGAGCTGGGTTAAGG	^16^
RPL 13 AB	Forward- CCTGTTTCCGTAGCCTCATG Reverse- AAGTACCAGGCAGTGACAG	^7^
Integrin α2	Forward- TGTCTGGCGTATAATGTTGGC Reverse- TGCTGTACTGAATACCCAAACTG	
Integrin α3	Forward- TGCCCATCGGTACACCAAG Reverse- ATTGCCACGCACATAGCACT	
Integrin α4	Forward- AACCGGGCACTCCTACAAC Reverse- CACCACCGAGTAGCCAAACAG	
Integrin α5	Forward- TGCAGTGGTTCGGAGCAAC Reverse- TTTTCTGTGCGCCAGCTATAC	
Integrin αV	Forward- AAAGACCGTTGAGTATGCTCCA Reverse- ATGCTGAATCCTCCTTGACAAAA	
Integrin β1	Forward- TGGTCAGCAACGCATATCTGG Reverse- GATCCACAAACCGCAACCT	

### Western blot analysis

Whole cell lysates were collected at the end of 7 and 14 days and subjected to western blot analysis. Briefly, the proteins were electroblotted onto a PVDF membrane from the SDS-PAGE gel and incubated with primary antibody overnight at 4 °C, followed by secondary antibody for 1 h at room temperature. Protein of interest was detected using ECL kit (WESTAR SUPERNOVA, Cyanagen, Bologna, Italy). ImageLab software version 4.5 was used for quantification.

### Animal studies

#### Animals and surgical procedure

Male albino Wistar rats were procured from NIN (National Institute of Nutrition), Hyderabad, each weighing approximately 250 g. Approval for animal experimental procedures was obtained from the Institutional Animal Ethics Committee, Kovai Pharmacy College, Coimbatore, India. The surgical procedures were carried out according to the Institutional Guidelines and Regulations for the Care and Use of Laboratory Animals (IAEC No: KMCRET/Ph.D/06/2018–19). Animals were anesthetized prior to surgery using 5% isoflurane and a right tibial perforation of 3 mm was made using a dental bur. Constant saline irrigation (0.9% NaCl) was provided throughout the procedure as described previously^34–36^. Group 1 defects were left untreated/unfilled, group 2 was filled with Gel/nHAp, and group 3 was filled with Gel/nHAp/20 μM DM scaffold. The animals were maintained for 4 weeks and 8 weeks followed by euthanasia by anesthesia overdose. Eight animals were used for each group and at each time point (3 animals for X-ray imaging, 1 for SEM-EDAX, and 2 for histological staining). Rat tibia was removed and radiographed followed by 10% formalin fixing for 48 h for further analyses^34–36^. Bone mineral density was calculated using ImageJ software^37^.

### Histological processing

The tibial bone section containing the defect/scaffold implants were dissected and they were subjected to histological analyses. Hematoxylin and Eosin staining (H&E) and Masson’s Trichome staining (MTS) were performed individually, as described previously^36^.

### SEM and EDAX analyses

Calcified sections of bone were analyzed for the implant-tissue interface mineralization using SEM and EDAX analysis. Bone sections were sputter coated with gold and visualized in a HR-SEM Quanta 200 FEG instrument (FEI, Eindhoven, Netherlands)^36^.

### Statistical analysis

All experiments were carried out in triplicates and the results are represented as mean ± SD. Statistical analysis was done using one-way ANOVA and student’s *t*-test. A ‘p’ value less than 0.05 is considered significantly different with respect to control.

## Results and Discussion

### Physiochemical characterization Gel/nHAp/DM scaffolds

Growth factor (GF)-incorporated biomaterials are considered a plausible alternative to autografts for bone regeneration. Several inadequacies, such as lesser stability and high cost, lead to a need to find an alternative for GF that has a better half-life, bioavailability, and low cost^3,38,39^. There are reports indicating that phytocompounds with anti-diabetic properties have a potential towards enhanced osteogenic effects in pre-osteoblastic and osteoblastic cells^40–43^. Due to their non-water-soluble properties, their bioavailability is limited. To enhance the bioavailability of the phytomolecule DM, and to determine its osteogenic potential, we utilized a Gel/nHAp scaffold. The main reason behind using the Gel/nHAp scaffold in this study was its close relation to the components in naturally occurring bone^28,44^. Gel can provide a swelling nature to the scaffold along with enhancing cell attachment, whereas nHAp plays a major role in protein adsorption, mechanical strength, bio-mineralization, osteo-conduction, and osteo-integration^23,34,35^. Cross-linkers can provide a controlled degradation to the scaffold and, thus mediating a sustained drug delivery^7,11^.

**Figure 1:**
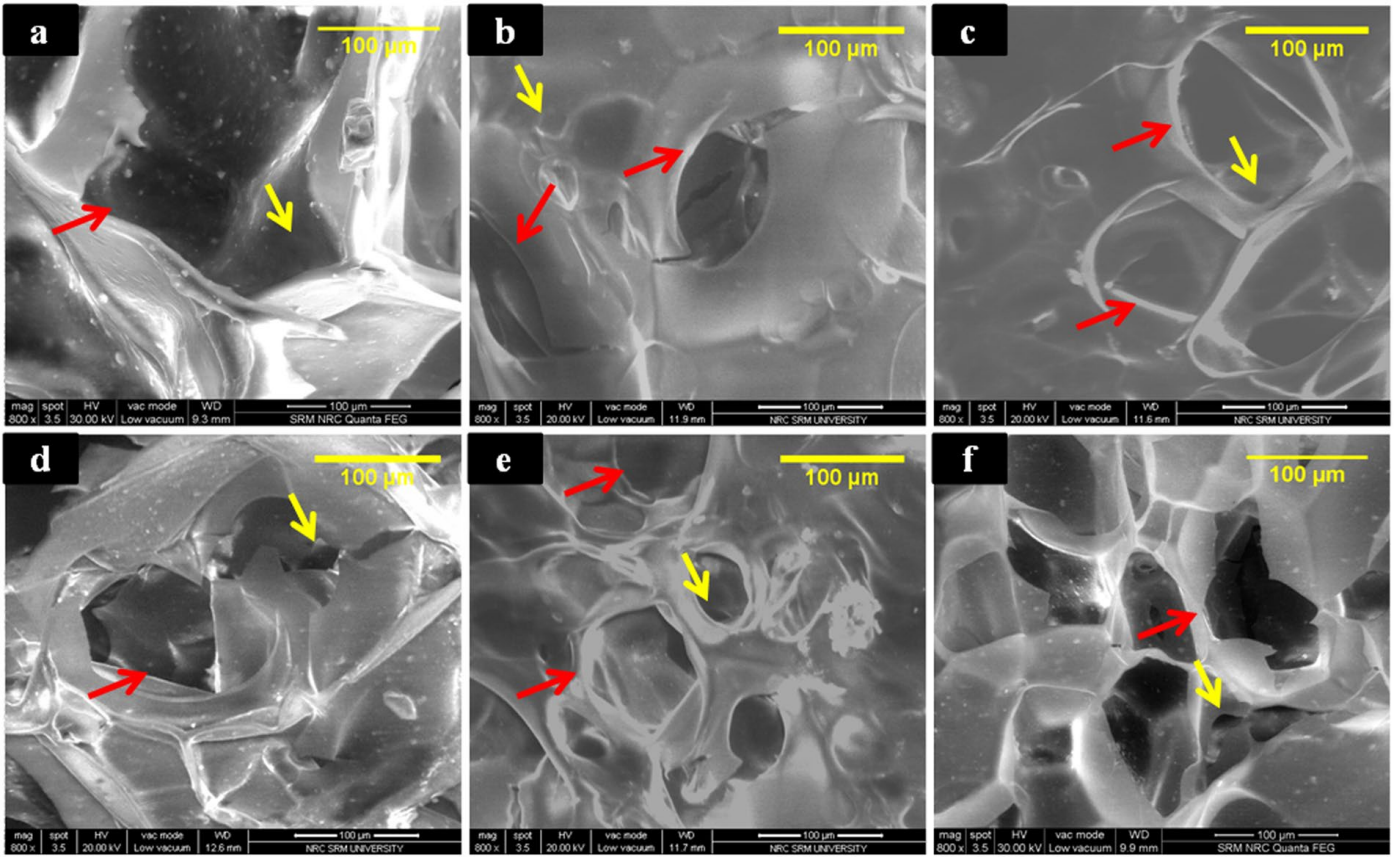
SEM images of biocomposite scaffold. (**a**–**f**) Represent the surface topography of Gel/nHAp, Gel/nHAp/20 µM DM, Gel/nHAp/40 µM DM, Gel/nHAp/60 µM DM, Gel/nHAp/80 µM DM, and Gel/nHAp/100 µM DM, respectively. (Red and yellow arrows indicate macro and micro interconnected pores, respectively).

Gel/nHAp/DM biocomposite scaffold was prepared using the lyophilization technique and physicochemical characterization was carried out. SEM analysis was performed to probe the external surface morphology, pore structure, and size of the prepared biocomposite scaffold. Macro and micro interconnected porous structures were found in the SEM images of the biocomposite scaffold and had the pore size ranging from 80 to 100 µm, which could support initial cell infiltration and nutrient exchange^45^. Addition of DM in varying concentrations (0, 20, 40, 80, and 100 µM) did not alter the pore size of the scaffold, indicating that DM does not have a role to play in the physical structure of the scaffold (Fig. 1). The analysis of the elemental composition of the fabricated scaffold was identified using EDS, and a Ca/P ratio was in the range from 1.4–2.0 (S. Fig. 1).

**Figure 2:**
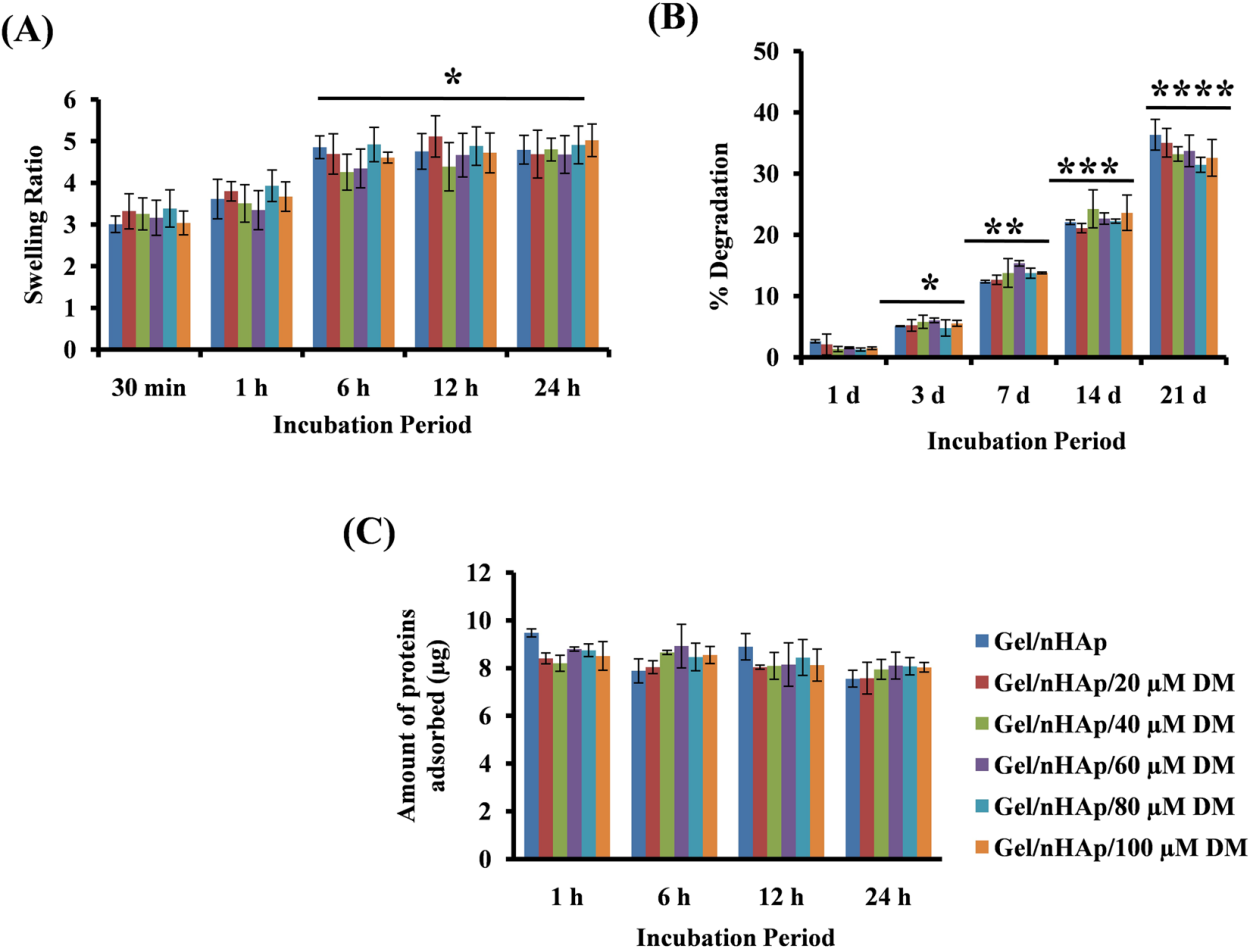
Swelling, degradation, and protein adsorption of scaffold. (**A**) Swelling ratio of the scaffolds incubated in 1X PBS after 30 min, 1, 6, 12, and 24 h. * indicates a significant increase in swelling ratio of respective scaffolds with respect to 30 min. (**B**) Percentage degradation of scaffold after 1, 3, 7, 14, and 21 days of incubation. * indicates significant increase with respect to 1 day, ** represents significant increase with respect to 3 days, *** represents significant increase with respect to 7 days, **** represents significant increase with respect to 14 days Gel/nHAp scaffold. (**C**) Amount of protein adsorbed on to the scaffold after incubating in 1% FBS containing DMEM after 1, 6, 12, and 24 h incubation.

FTIR analysis of individual compounds revealed the respective characteristic peaks corresponding to their functional groups (S. Fig. 2A). nHAp showed a broad spectrum peak at 3443 cm^−1^ corresponding to the –OH stretch, and the peaks at 1060, 961.7, 493.885, 572.651, 603.294 cm^−1^ were absorption bands of PO_4_^2−^. DM showed a –OH stretch at 3466 cm^−1^, absorption bands at 1611 and 1659 cm^−1^ corresponded to the aromatic C=C bending, a band at 2923 cm^−1^ indicated the alkyl –CH stretch, and a band at 1068 cm^−1^ corresponded to C=C bond. A Gel amide bond was observed at 1640 cm^−1^. All the major characteristic peaks of individual compounds were present in the composite scaffold and the disappearance of the nHAp peak at 1060 cm^−1^ might be due to the modification that occurred during the chemical linkage between –COO group of Gel with Ca^2+^ in the interfacial surface of nHAp via the crosslinker EDC^46^. An increase in peak intensity by two characteristic peaks corresponding to DM at 1639 and 1059 cm^−1^ with an increase in DM concentration in the scaffold indicated the successful incorporation of DM into the Gel/nHAp scaffold. Powder XRD can provide evidence of any change in crystalline nature due to host-guest interaction. In XRD analysis, the Gel possessed a semi-crystalline nature whereas nHAp and DM were crystalline in nature (S. Fig. 2B). DM showed several peaks between 0° and 30° indicating its high crystallinity whereas, XRD diffraction peaks of Gel/nHAp/DM composites showed the characteristic peaks of nHAp alone at 31.8°, 32.2°, and 32.9° (JCPDS-09-0432). Peaks of DM were not observed in the scaffold, which might be due to a possible reduction in crystallinity of DM upon addition to Gel.

### Swelling, degradation and protein adsorption

Upon incubation of the scaffold in 1X PBS, an early increase in swelling was observed in all groups, irrespective of the presence or absence of DM until 1 h and after the swelling ratio was stabilized and no further increase or decrease was observed. The structural integrity was also retained until the end of the study (Fig. 2A). The degradation result showed that the scaffold has a lower degradation rate in the initial time period and an average of 45% degradation was observed at the end of 21 days (Fig. 2B). Instant protein adsorption was also seen when the scaffold was incubated with 1% FBS containing media, and a further increase in time periods had no effect on the amount of protein adsorbed. Thus, the scaffold had swelling, degradation, and protein adsorption potential, which are required features for a bone regenerative scaffold and these properties were not altered by the presence of DM in the scaffold (Fig. 2C).

### Bio-mineralization and drug release studies

**Figure 3:**
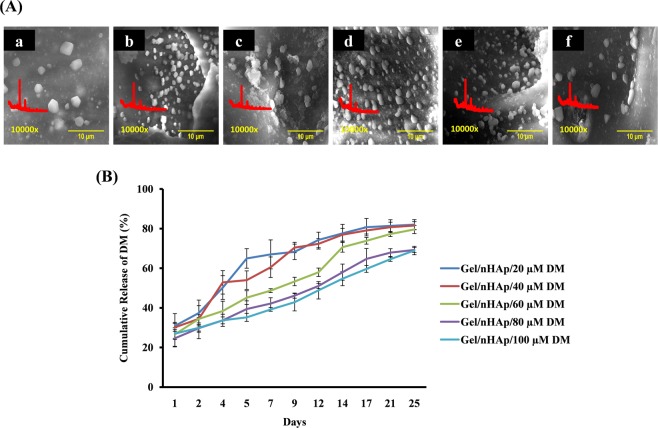
Bio-mineralization and DM release studies of biocomposite scaffold. (**A**) a–f represent SEM images and XRD spectra of bio-mineralized scaffold Gel/nHAp, Gel/nHAp/20 µM DM, Gel/nHAp/40 µM DM, Gel/nHAp/60 µM DM, Gel/nHAp/80 µM DM, and Gel/nHAp/100 µM DM, respectively after 14 days of incubation in SBF at 37 °C. (**B**) Cumulative release percentage of DM from scaffold after incubating it in 1X PBS at 37 °C for 25 days.

Bio-mineralization is the ability of the scaffold to form apatite crystal deposits onto its surface from the surrounding fluids. Simulated body fluids (SBF) have a similar ionic concentration found in human body fluids^47^. SEM images revealed the formation of crystal deposits onto the scaffold surface after 7 days of incubation in SBF (S. Fig. 3) and the crystal size was found to increase with an increase in incubation time up to 14 days (Fig. 3A). The XRD analysis of the bio-mineralized scaffold showed the characteristic peaks of apatite, which were in concordance with JCPDS No. 09-0432 of HAp. The elemental Ca/P ratio of biomineralized scaffolds was in the range of 1.6–2.2. Thus, the crystals formed on the surface of the scaffold were of HAp and the addition of DM had no effect on bio-mineralization potential of the scaffold.

A sustained and prolonged release of the drug from the scaffold is a major aspect of bone healing, as it ensures the presence of the drug in the defective area until the end of the healing period and also ensures drug delivery to the defect site without undergoing renal clearance. Reports are indicating that hydrophilic polymers such as cyclodextrin, polyethylene glycol 6000, and hydroxypropyl methyl cellulose with DM reduced the crystallinity of DM and improved *in vitro* dissolution^24,25^. In this study, we used a hydrophilic and well-established drug carrier molecule, Gel for DM release^26^. DM was released in a sustained and prolonged fashion from the scaffold, and the burst release of DM on the first day might be due to the compound being on the surface of the scaffold (Fig. 3B). The release of DM from scaffold was determined from 24 h to 25 days due to the nature of *in vitro* study in cell culture model system and *in vivo* study in the rat model system. At the end of 25 days, the samples were collected and subjected to HPLC analysis and the results confirmed the presence of DM in the supernatant with a retention time of 19.810 min compared to the standard.

### Cyto-compatibility of Gel/nHAp/DM scaffold

**Figure 4:**
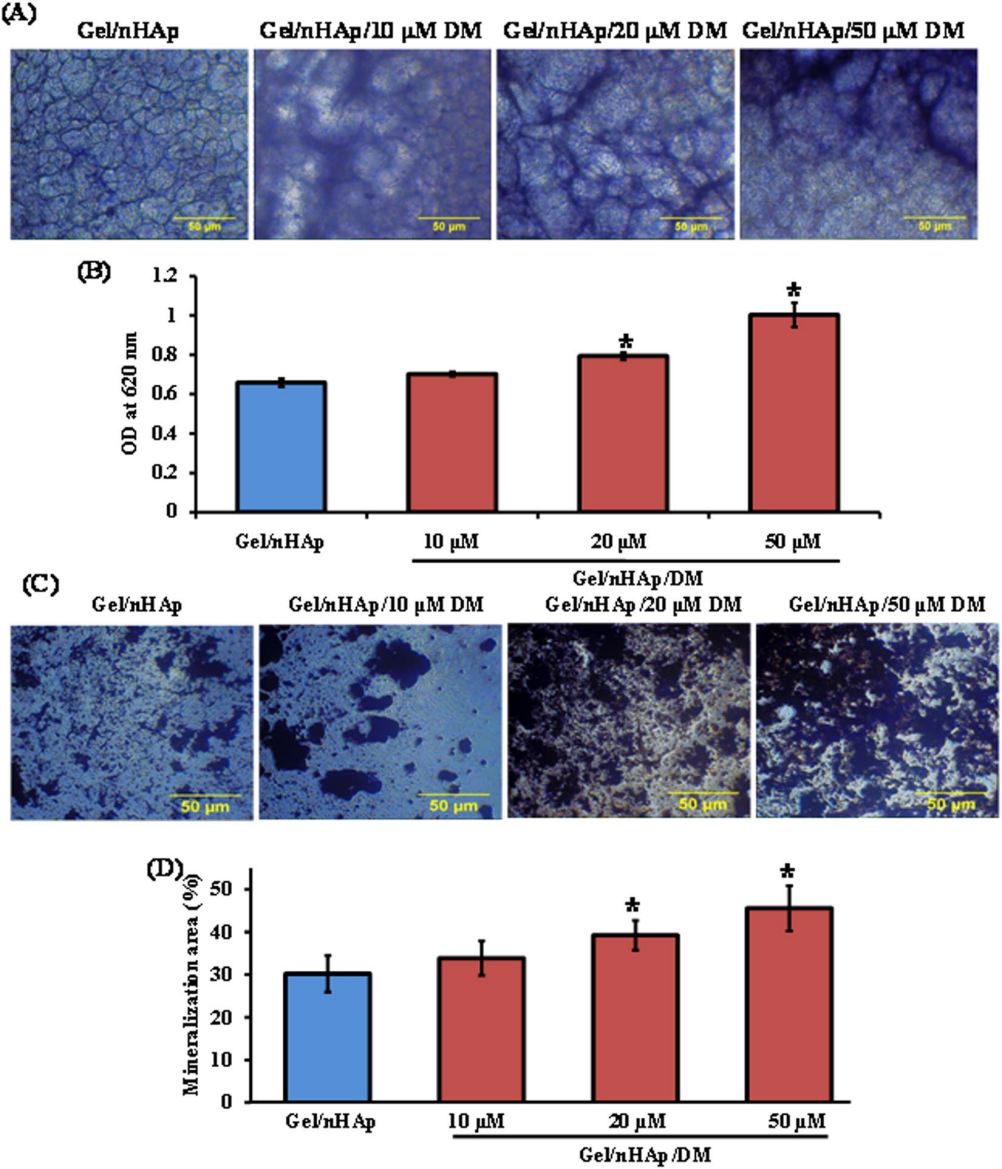
Effect of DM in osteoblast differentiation at cellular level. C3H10T1/2 cells were seeded on to scaffold and cultured for 7 and 14 days for ALP staining and von Kossa staining, respectively. (**A**,**B**) Represent microscopic image and quantification of ALP stained areas, respectively. (**C**,**D**) Represent microscopic image and quantification of the area of mineralization, respectively. * indicates a significant increase compared to Gel/nHAp (*p* < *0.05*).

To determine the osteo-inductive potential of DM, we initially determined the expression of Runx2 in C3H10T1/2 cells. Cells were treated with varying concentrations (10–100 µM) of DM and Runx2 mRNA expression was determined by RT-qPCR. The results showed an optimum up-regulation of Runx2 gene expression in 20 µM DM-treated C3H10T1/2 cells (result not shown). In the physiochemical and material characterization studies (Figs 1, 2), the addition of DM, even at higher concentrations, had no role on altering scaffold characteristics. Hence, we selected only 3 concentrations of DM, i.e. 10, 20, and 50 µM, to incorporate into Gel/nHAp scaffolds for further studies. Cyto-compatibility is considered a prerequisite for any compound to be used for clinical applications. Gel/nHAp scaffolds were already proven to be cyto-compatible^28^, thus alteration in cell viability due to the addition of DM to the scaffold was studied. Since the release of DM from scaffold was in the average of 40% after 72 h of incubation (Fig. 3B), we checked the toxicity of DM towards cells for 72 h. The result showed no change in cell viability by these scaffolds (S. Fig. 4A). In FDA assays, the conversion of non-fluorescent FDA to fluorescein by active cell esterases was determined^7^. The morphological evaluation assessment using FDA staining showed cells adhered to the scaffold with a flattened morphology and well-defined cytoplasmic extensions (S. Fig. 4B) proving the scaffolds to be non-toxic to mMSCs.

### Gel/nHAp/DM scaffold enhanced osteoblast differentiation

To determine the osteogenesis potential of DM at the cellular and molecular levels, C3H10T1/2 cells were seeded onto the scaffold and cultured for a time period of 7 and 14 days. At the cellular level, cells seeded onto DM-containing scaffold showed a higher ALP activity compared to control after 7 days (Fig. 4A,B). ALP activity is not confined to osteoblast cells, and mineralization potential is considered the key factor for successful bone regeneration. Thus, von Kossa staining was performed to determine the calcium phosphate deposition at the end of 14 days. Silver nitrate reacted with the calcium phosphate deposits made by the differentiated cells and formed silver phosphate, which when irradiated with UV formed metallic silver dark brown precipitates. The result showed that the addition of DM into scaffold enhanced calcium phosphate deposits by mMSCs (Fig. 4C,D).

**Figure 5:**
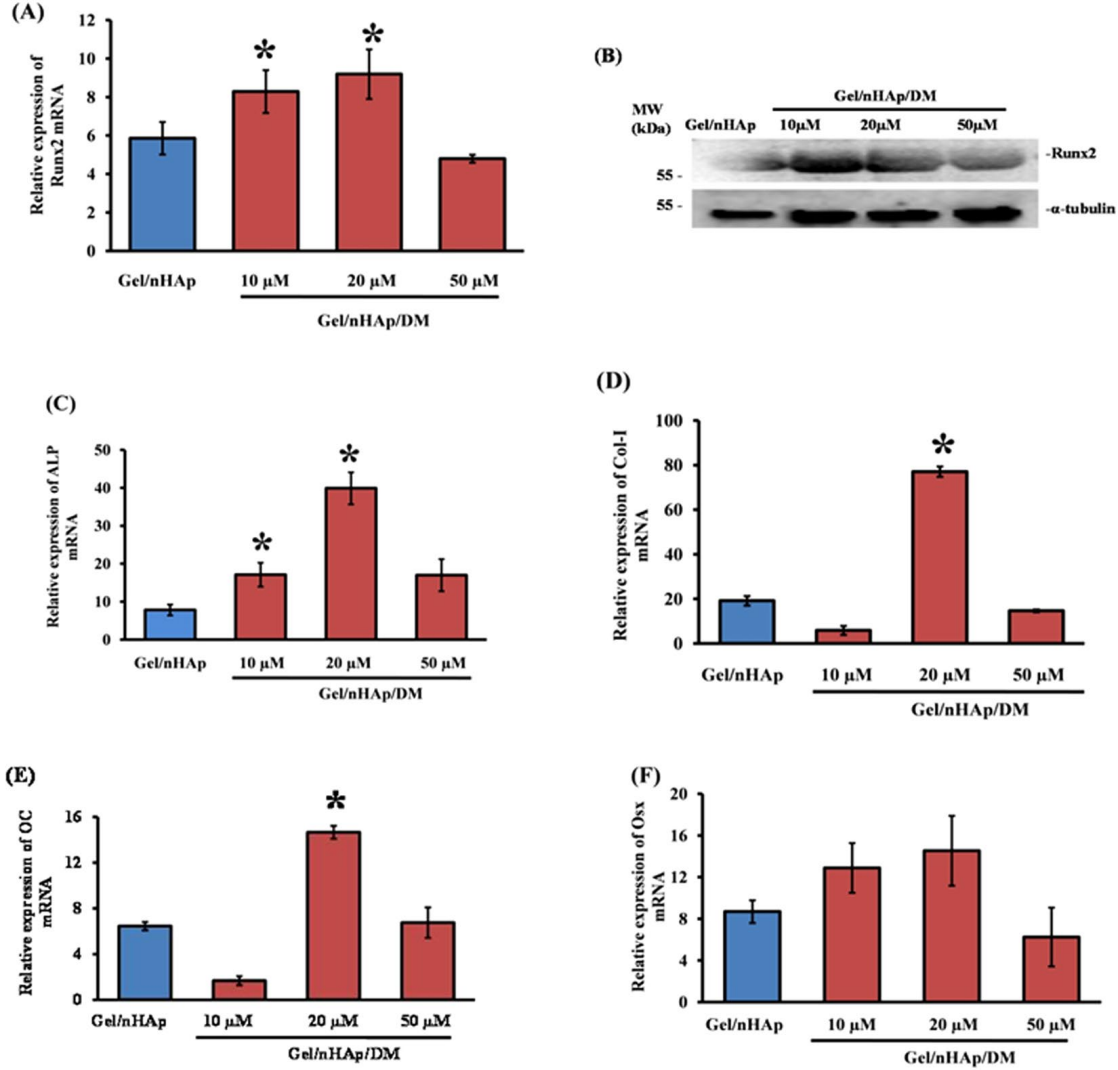
Effect of DM in osteoblast differentiation at molecular level. C3H10T1/2 cells were seeded on to DM-containing scaffolds and cultured for 7 days. Total RNA and protein were isolated and subjected to the analyses of RT-qPCR and western blot, respectively. (**A**) Represents Runx2 mRNA expression. (**B**) Represents Runx2 protein expression (full-length blots are presented in the Supplementary Figures). (**C**–**F**) Represent the mRNA expression of ALP, Col-I, OC, and Osx, respectively. *Indicates a significant increase compared to Gel/nHAp (*p* < *0.05*).

To confirm the osteogenic effect of DM at the molecular level, C3H10T1/2 cells were seeded onto the scaffold and the expression of osteoblast differentiation marker genes was determined using RT-qPCR and western blot analyses. Differentiation of MSCs towards osteoblast is mediated by two main transcription factors namely, Runx2 and Osterix, and several other osteogenic markers, such as ALP, Col-I, osteocalcin (OC), etc. ALP and OC are responsible for calcium phosphate or apatite formation in the osteoblastic cells where the former helps in conversion of pyrophosphate, which inhibits HAp formation in osteoblasts and the latter is responsible for apatite deposition on to the matrix^7,48,49^. Runx2 acts as a molecular switch in the conversion of mesenchymal and pre-osteoblastic cells towards osteoblast and Osterix is responsible for osteoblast maturation^16,50–52^. Runx2 expression was up-regulated when cells were grown in the DM-containing scaffold for 7 (Fig. 5A,B; full-length blots are presented in the Supplementary Figures) and 14 days (S. Fig. 5A–C; full-length blots are presented in the Supplementary Figures) at both the mRNA and protein level. The expression of osteoblast marker genes also showed an increase in the expression of ALP (Fig. 5C), Col-1 (Fig. 5D), and OC (Fig. 5E) after 7 days of incubation; whereas there was no significant difference observed in Osterix (Fig. 5F) expression. After 14 days incubation, the mRNA expression of ALP (S. Fig. 5D), Col-I (S. Fig. 5E) and Osterix (S. Fig. 5F) increased in the cells with respect to control. Most of the genes that participate in osteoblast differentiation as shown above were up-regulated by Gel/nHAp scaffold incorporated with 20 µM DM. When cells were incubated with scaffold containing a higher concentration of 50 μM DM, the expression of Runx2 and other osteoblast marker genes decreased (Fig. 5), which might be due to the negative feedback regulation of Runx2 in the cells^53,54^.

### Adhesion-mediated osteogenesis

**Figure 6:**
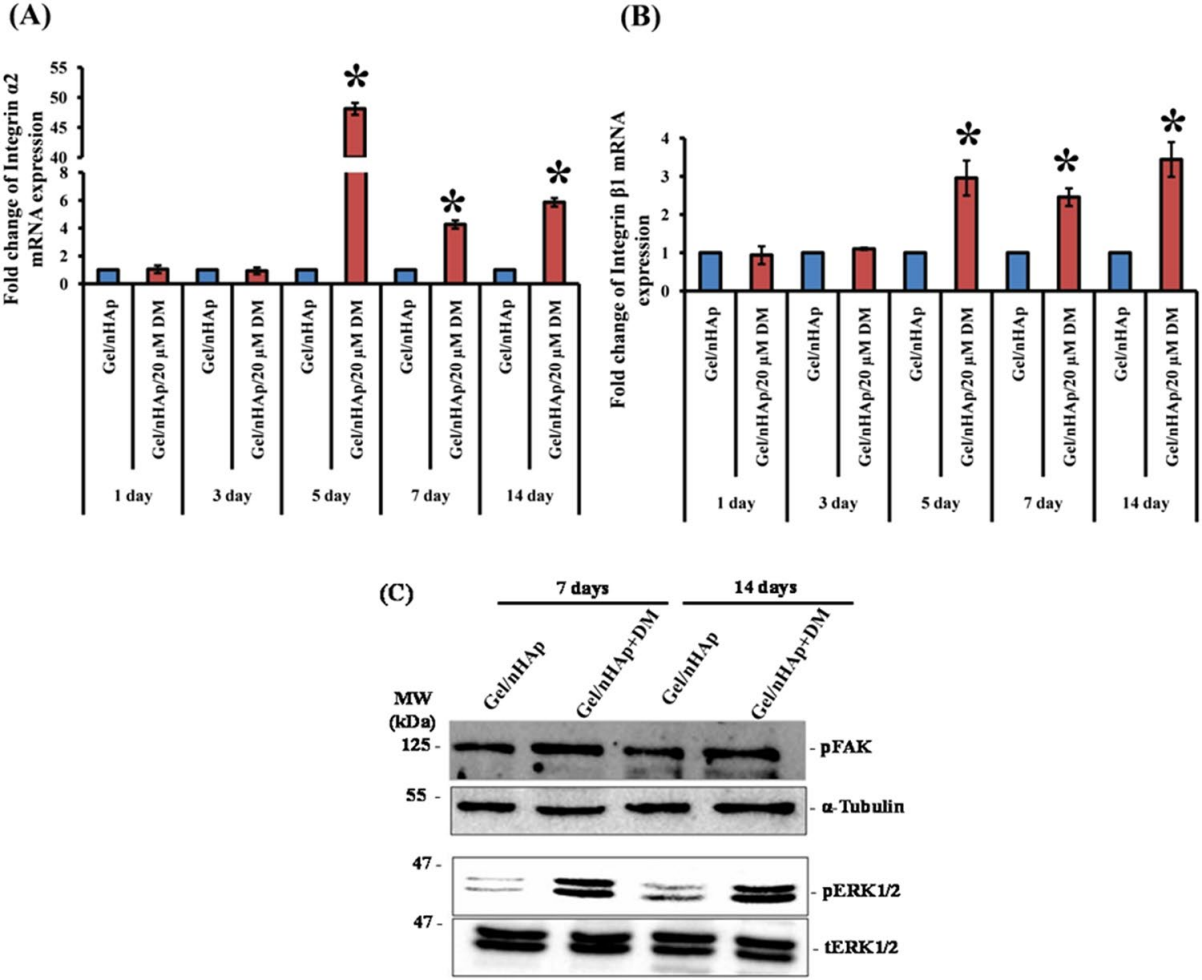
Effect of DM in cell integrin-mediated activation of FAK and ERK. C3H10T1/2 cells were seeded on to scaffold and cultured for 1, 3, 5, 7, and 14 days followed by RNA isolation, cDNA synthesis, and qPCR analysis. (**A**,**B**) Represent the mRNA expression of integrin α2 and β1. * indicates a significant increase compared to respective Gel/nHAp (*p* < *0.05*). (**C**) Represents protein expression of pFAK, pERK1/2, and tERK1/2 (full-length blots are represented in the Supplementary Figures). α-tubulin was used as internal control for normalization. p: phosphorylated; t: total.

From the above results (Figs 4, 5), it is evident that the Gel/nHAp scaffold loaded with 20 μM DM had the maximal effect on promoting osteoblast differentiation and hence, DM loaded with 20 μM in Gel/nHAp scaffold was used for further studies. Cell adhesion to the matrix is mediated by various integrins, a family of transmembrane proteins, and integrins play a major role in cell differentiation^48^. Our previous result showed that DM promoted collagen expression (Fig. 5D) and this would also be one of the prime ligands for integrin binding. Thus, we determined the expression of osteoblast-specific integrins such as α1, α2, α3, α4, α5, αV, and β1 after culturing mMSCs on to the scaffold at various time periods of 1, 3, 5, 7, and 14 days. The gene expression analysis showed that an enhanced level of α2 (Fig. 6A), α3 (S. Fig. 6A), α5 (S. Fig. 6B), and β1 (Fig. 6B) at 5, 7, and 14 days of incubation, whereas αV remained unaltered (S. Fig. 6C).

To further lineate the integrin-mediated signaling pathway involved in the promotion of osteogenesis by DM, we determined the activation of FAK and ERK in mMSCs. The addition of DM in the Gel/nHAp scaffold led to activation of FAK and ERK in terms of phosphorylation at both 7 and 14 days in the mMSCs (Fig. 6C). Integrin α2β1 aggregation was found to be essential for osteogenic cell differentiation and α5 was responsible for osteoblast differentiation and maturation in mesenchymal skeletal cells. These integrins corresponded to ligands such as collagen and fibronectin^48,50^. Col-I was found to be essential for osteoblast differentiation and its interaction with α2β1 in cells helped in the development of the osteoblastic phenotype^50,55^. It has also been shown that Runx2 activation was mediated by collagen-integrin interaction via ERK, which is a downstream signal of FAK^50,55,56^. The role of phytomolecules in integrin-mediated signaling for osteogenesis has not been explored yet; thus, in this study, we dissected the differential expression of integrins and activation of their down-stream signaling pathways by mMSCs cultured onto Gel/nHAp/20 μM DM scaffold. It has been shown that pFAK associated with src and Grb2 activated ERK, a member of the MAPK family via Ras^50,55,57^. There are reports indicating the importance of MAPK signaling (ERK, p38 MAPK) and Runx2 expression/activation^56,58–61^. Runx2 was required for the expression of ALP, Col-I, and OC, which are the markers for osteoblast differentiation^23,59,62^. Our results showed that there was activation of ERK by mMSCs cultured onto Gel/nHAp/20 µM DM scaffold suggesting that pFAK triggered activation of pERK (Fig. 6C), followed by Runx2 (Fig. 5A,B) and collagen (Fig. 5D) expression. Thus, we hypothesized that collagen deposition on to the surface of the Gel/nHAp/20 µM DM scaffold by the cells may also lead to an increase in the differential expression level of integrins, resulting in cell adhesion-mediated signaling for osteogenesis (Fig. 8). A similar mode of action with the osteogenic stimulant ascorbic acid was shown for osteogenesis^12,13^.

### Gel/nHAp/DM scaffold in bone formation *in vivo*

**Figure 7:**
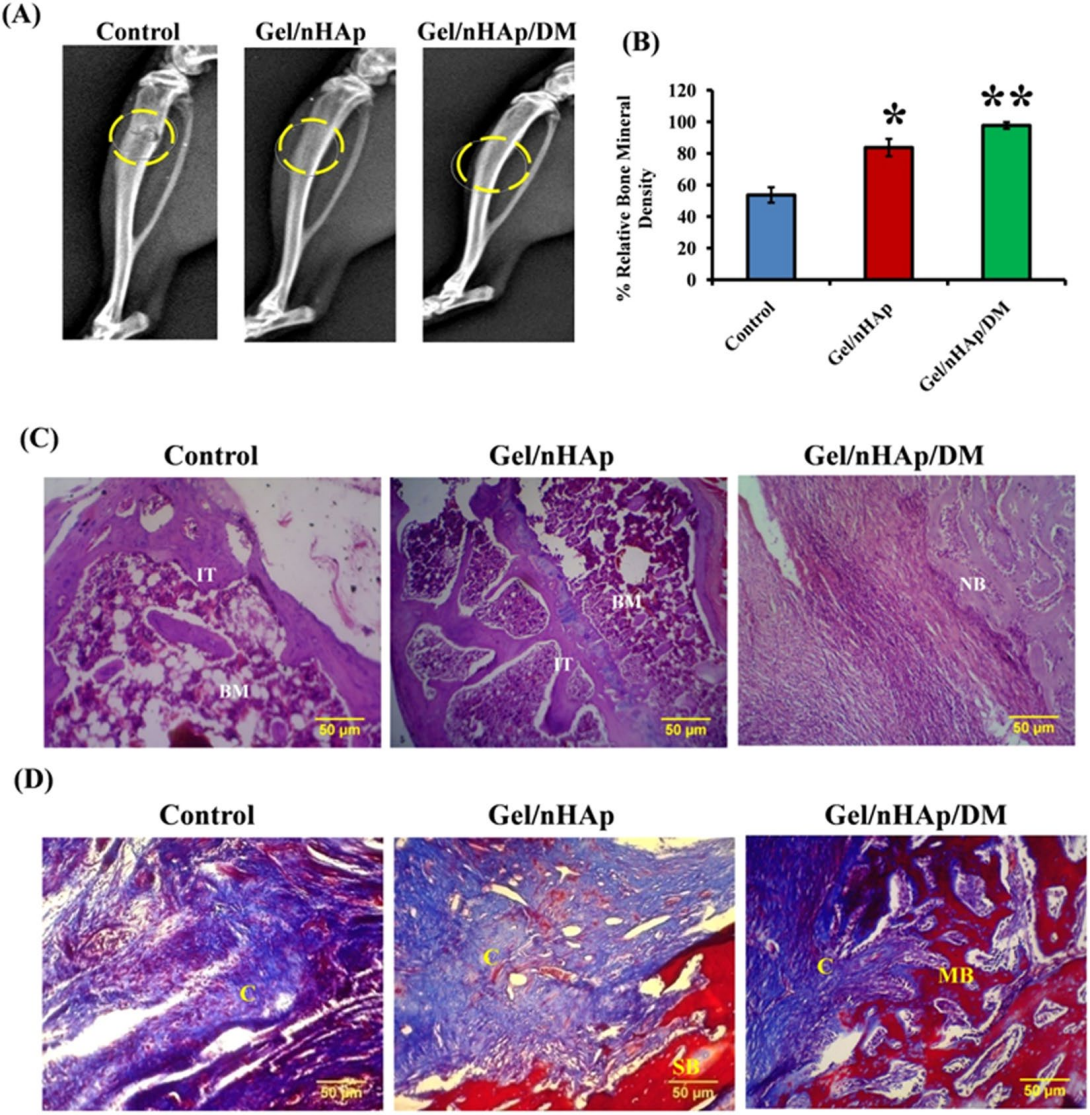
Effect of DM in bone formation *in vivo*. (**A**) X-ray images of the tibial defect after 8 weeks implantation with control, Gel/nHAp and Gel/nHAp/DM scaffolds. (**B**) Quantification graph of BMD using ImageJ software. * indicates significant increase compared to control (*p* < *0.05*) and ** represents a significant increase compared to control and Gel/nHAp (*p* < *0.05*). (**C**,**D**) H and E staining and Masson’s trichome staining of decalcified new bone areas. IT- immature trabeculae, BM – bone marrow space, NB – new bone, C – collagen, SB – surrounding bone, MB – mature bone.

**Figure 8:**
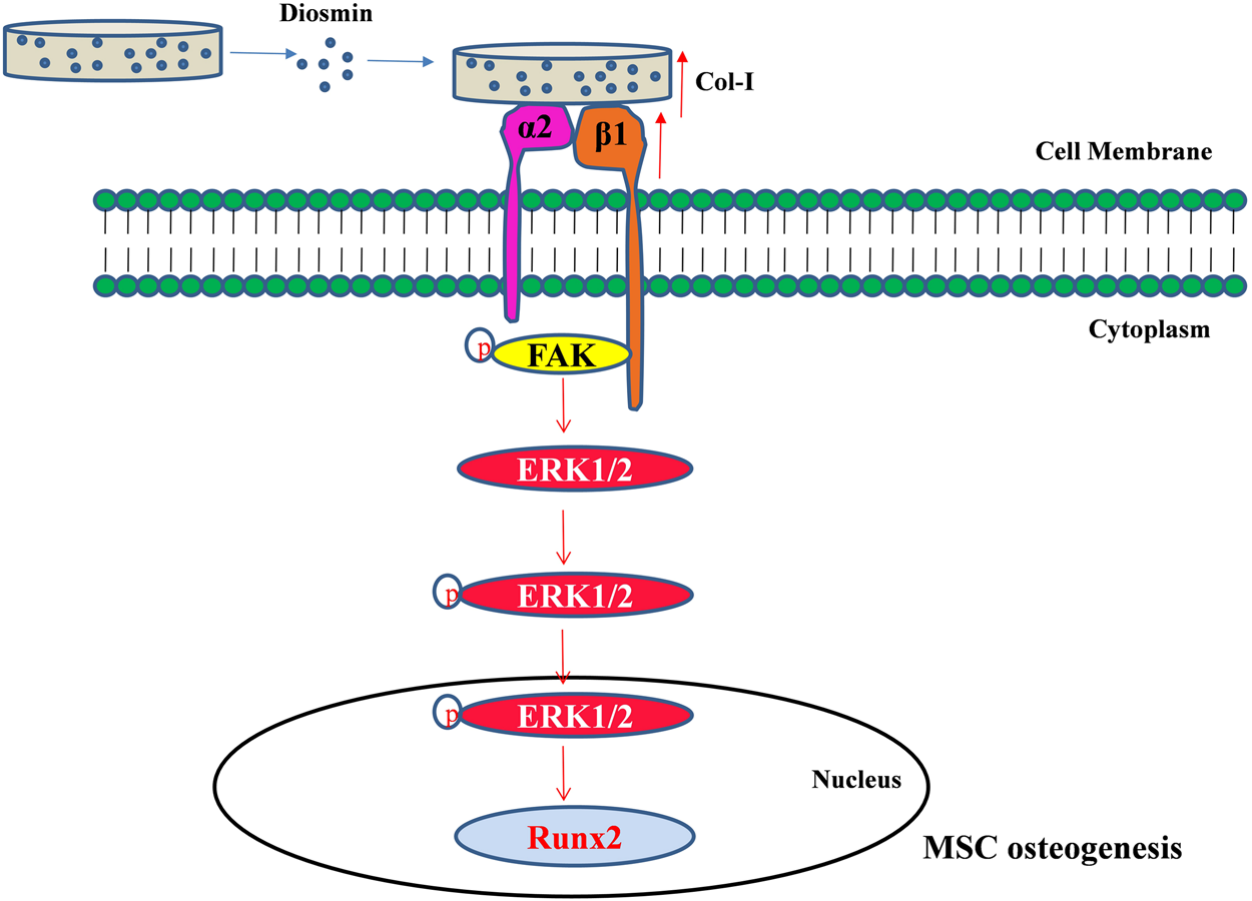
A schematic diagram of the DM effect on cell adhesion-mediated activation of signaling and osteogenesis. DM increased the expression of Col-I, Integrin α2 and β1 expression, which led to activation of FAK, followed by ERK activation and Runx2 expression, resulting in mMSC differentiation towards osteoblast.

Inability to mimic an analogous success rate of *in vitro* model in an animal model is a major drawback of surface modified scaffold-mediated osteogenesis. To substantiate the osteogenic potential of Gel/nHAp/20 µM DM scaffold *in vitro*, we utilized the rat tibial bone defective model system. This system has been used in a number of studies and it has provided evidence for identifying the osteogenic potential of any biomaterials including drugs under living conditions^63–65^. Tibial bone defect model system also supports the evaluation of the regeneration potential of the scaffold at the load-bearing sites^64,65^. Thus, a 3 mm diameter defect was created on the rat tibia using a dental burr and filled with Gel/nHAp or Gel/nHAp/20 μM DM scaffold. The control group was left untreated. After 4 and 8 weeks of implantation, X-ray radiograph images were taken. There was a radiolucent space in the defective regions of both control and Gel/nHAp treated groups, whereas Gel/nHAp/20 μM DM implanted groups showed radio-opaque region confirming the formation of new bone (S. Fig. 7A). After 8 weeks of implantation, both of the scaffold treated groups showed radio-opaque regions while the control group remained radiolucent (Fig. 7A). There was a significant increase of BMD (bone mineral density) by Gel/nHAp and Gel/nHAp/20 μM DM scaffolds compared to control after 4 weeks (S. Fig. 7B) and 8 weeks (Fig. 7B) of implantation. Compared to control and Gel/nHAp scaffold, DM-containing Gel/nHAp scaffold had a further significant increase in BMD.

SEM and EDAX studies in the implanted regions showed smooth topographical features with more area of mineralization, which is similar to naturally occurring bone in Gel/nHAp/20 μM DM-treated groups whereas control and Gel/nHAp groups showed irregular surface topography and lesser Ca/P deposition (S. Figs 8, 9). The tibial sections were decalcified and analyzed by H & E and MTS staining. Decalcification of bone tissue was necessary for obtaining thin sections of bone tissue using microtome. Gel/nHAp-treated groups showed the formation of immature trabeculae with vacant bone marrow spaces in between, whereas there was formation of immature trabeculae with no vacant spaces in Gel/nHAp/20 μM DM-treated groups after 4 weeks of implantation. Control groups were devoid of trabeculae formation and more vacant spaces were observed (S. Fig. 10A). After 8 weeks, in the implantation of Gel/nHAp/20 μM DM-treated groups, there was mature new bone formation, whereas in Gel/nHAp-treated groups, the area was almost filled with an immature trabeculae region (Fig. 7C). These results were in concordance with the X-ray images obtained showing early healing of bone defect treated with DM containing scaffold. MTS images revealed an early induction of collagen deposition after 4 weeks in DM-treated groups with mature bone formation, whereas control and Gel/nHAp scaffold showed a lesser extent of collagen deposition (S. Fig. 10B). There was an increased collagen stained area in Gel/nHAp/20 μM DM-treated groups compared to control group and Gel/nHAp after 8 weeks implantation (Fig. 7D). Thus, the role of DM-loaded in Gel/nHAp scaffold in the promotion of bone regeneration *in vivo* after 8 weeks implantation was identified using the rat tibia bone defective model system (Fig. 7). Taken together of *in vitro* and The decrease in BMD is a major event in several age-related bone disorders such as osteoporosis, which leads to a higher risk of bone damage and reduced quality of living. Hence, a DM-loaded Gel/nHAp scaffold is a plausible alternative for scaffold incorporated with GF in bone regeneration and can be further studied for its possible effects in bone-related diseases, in which a decrease in bone mass or collagen content is a significant aspect.

## Conclusion

The current findings determined a role of DM in the Gel/nHAp scaffold for bone formation both *in vitro* and *in vivo*. Addition of DM had no effect on altering the physicochemical characteristics of Gel/nHAp scaffold, but enhanced the potential for osteoblast differentiation via up-regulation of osteoblast transcription factor, Runx2 and its differentiation marker genes. We also identified the cell surface integrin-mediated osteogenic potential of DM via the activation of FAK, ERK, and Runx2 expression (Fig. 8). A sustained and prolonged release of DM from the Gel/nHAp scaffold enhanced the bone healing effect, resulting in bone regeneration *in vivo*. Thus, the scaffold containing DM can be considered a potential candidate for bone tissue repair as an osteogenic substitute.

## Supplementary information

Supplementary Figures Revised

## Data Availability

The datasets generated during and/or analysed during the current study are available from the corresponding author on reasonable request.
