# Supplementary material for: Osteostimulatory effect of biocomposite scaffold containing phytomolecule diosmin by Integrin/FAK/ERK signaling pathway in mouse mesenchymal stem cells
Source: Sci Rep. 2019 Aug 15;9:11900. doi: 10.1038/s41598-019-48429-1 (PMC6695412; doi:10.1038/s41598-019-48429-1)
Supplement: Supplementary file 1 — Supplementary Figures Revised [file 41598_2019_48429_MOESM1_ESM.pdf]

Osteostimulatory effect of biocomposite scaffold containing phytomolecule diosmin by Integrin/FAK/ERK signaling pathway in mouse mesenchymal stem cells

S. Viji Chandran, M. Vairamani, and N. Selvamurugan

S. Figure 1

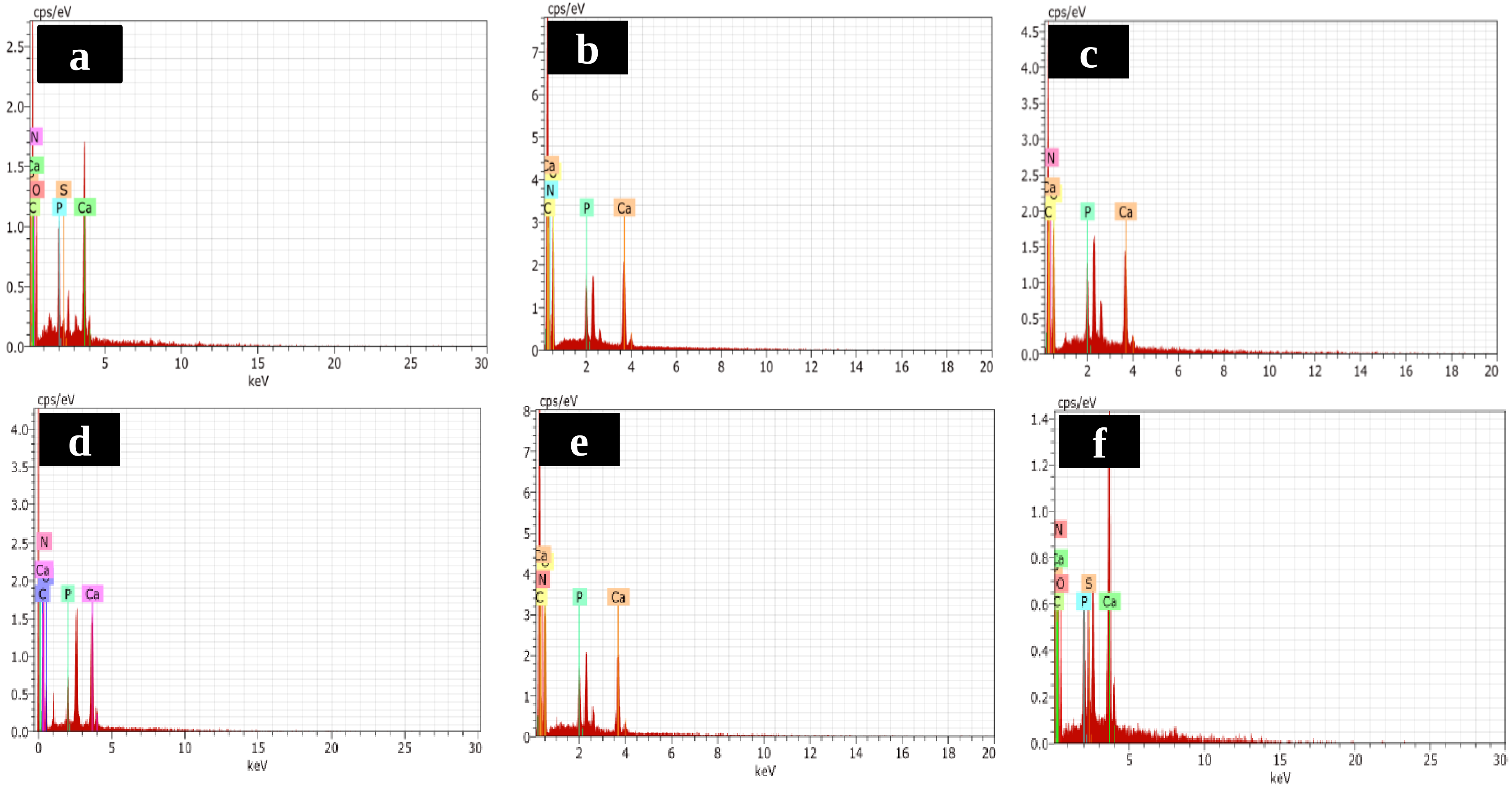

Energy dispersive X-ray spectroscopy. **(a-f)** represent the elemental composition of Gel/nHAp, Gel/nHAp/20 µM DM, Gel/nHAp/40 µM DM, Gel/nHAp/60 µM DM, Gel/nHAp/80 µM DM and Gel/nHAp/100 µM DM respectively.

S. Figure 2

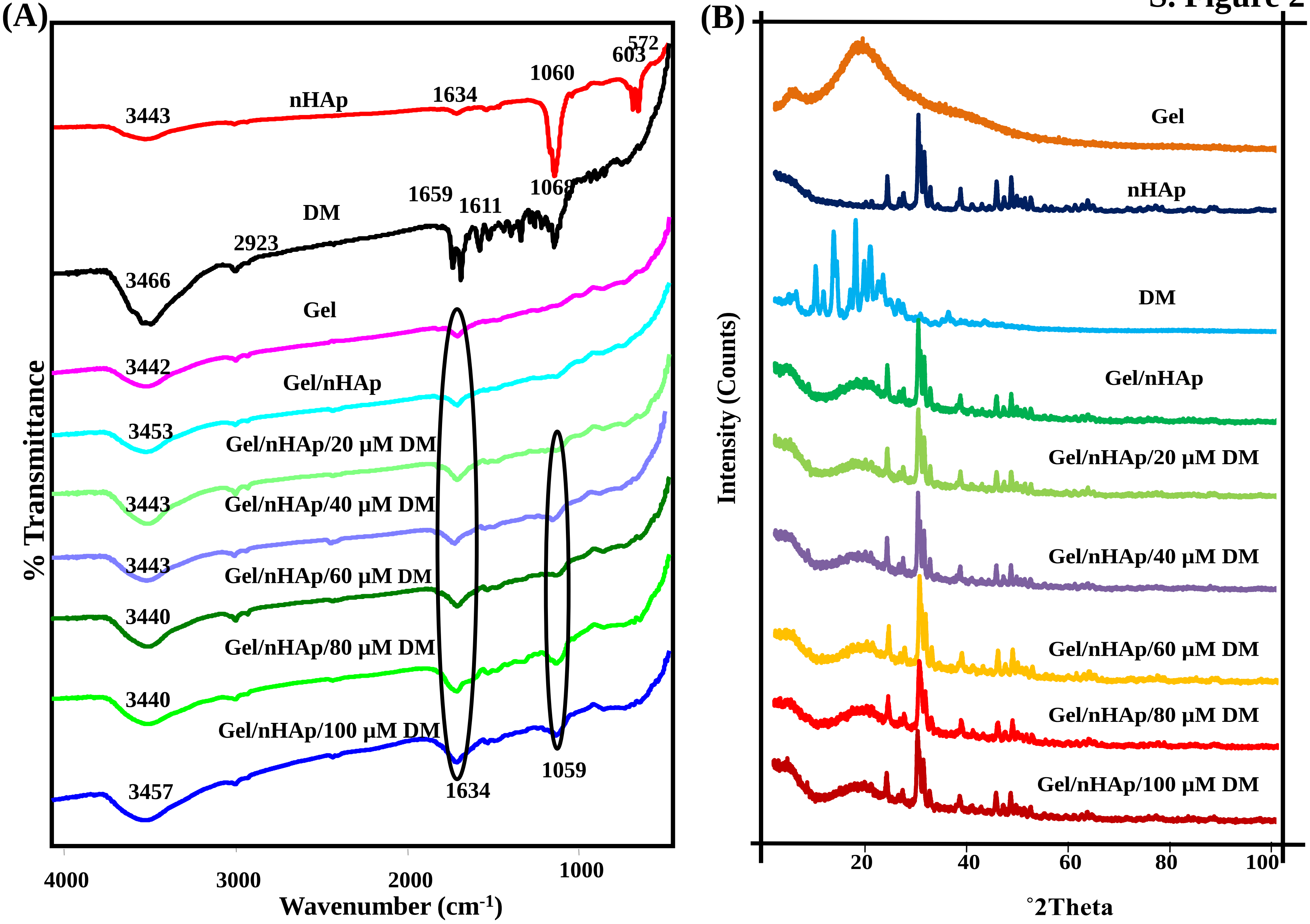

FT-IR and XRD analyses of scaffold. **(A)** FT-IR spectra of nHAp, DM, Gel, Gel/nHAp/DM scaffold, **(B)** XRD spectra of Gel, nHAp, DM, Gel/nHAp/DM

S. Figure 3

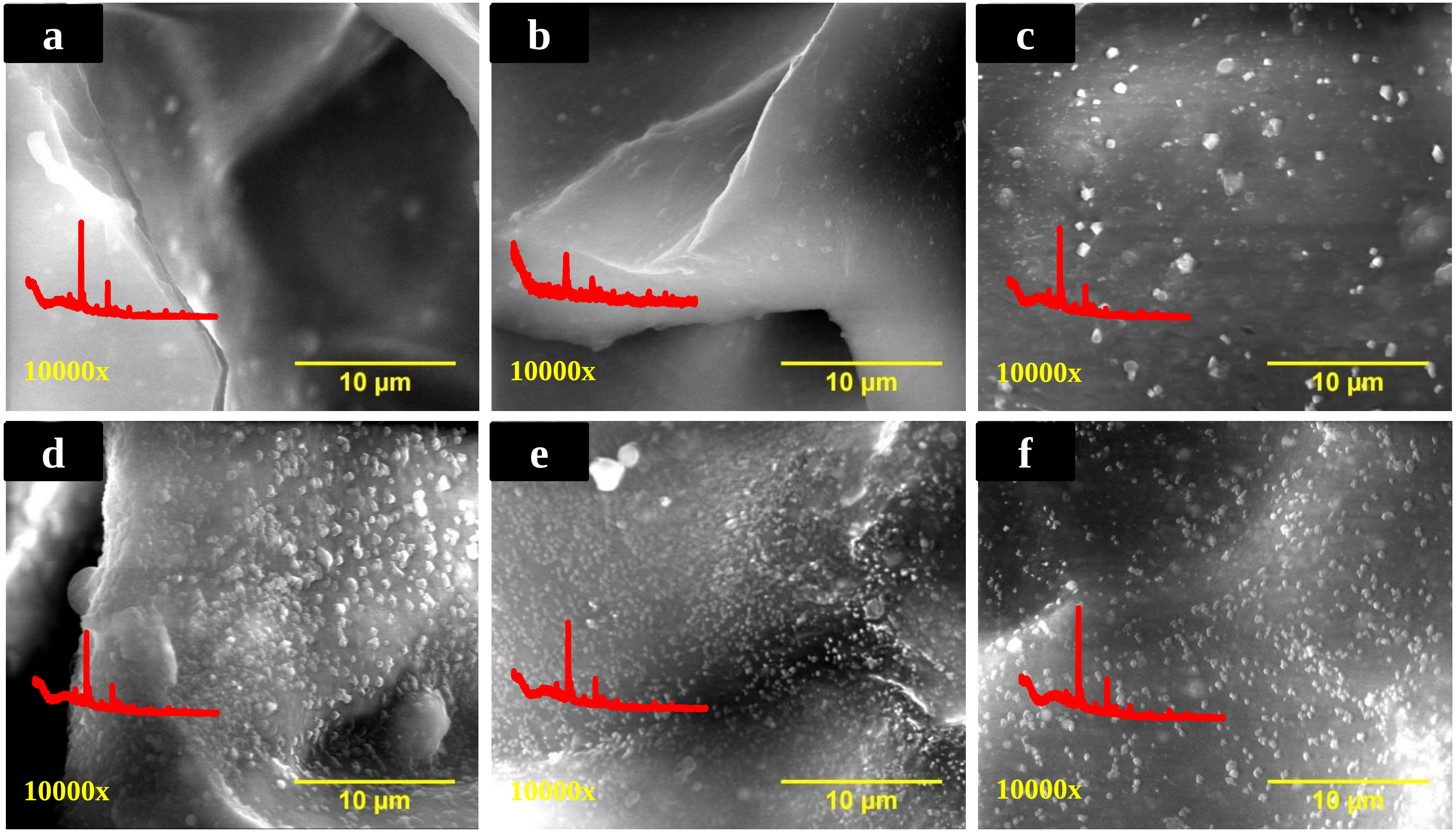

Biomineralization of scaffold. **(a-f)** represent the SEM images and XRD spectra of bio-mineralized scaffolds, Gel/nHAp, Gel/nHAp/20 µM DM, Gel/nHAp/40 µM DM, Gel/nHAp/60 µM DM, Gel/nHAp/80 µM DM and Gel/nHAp/100 µM DM, respectively after 7 d incubation in SBF at 37˚C.

S. Figure 4

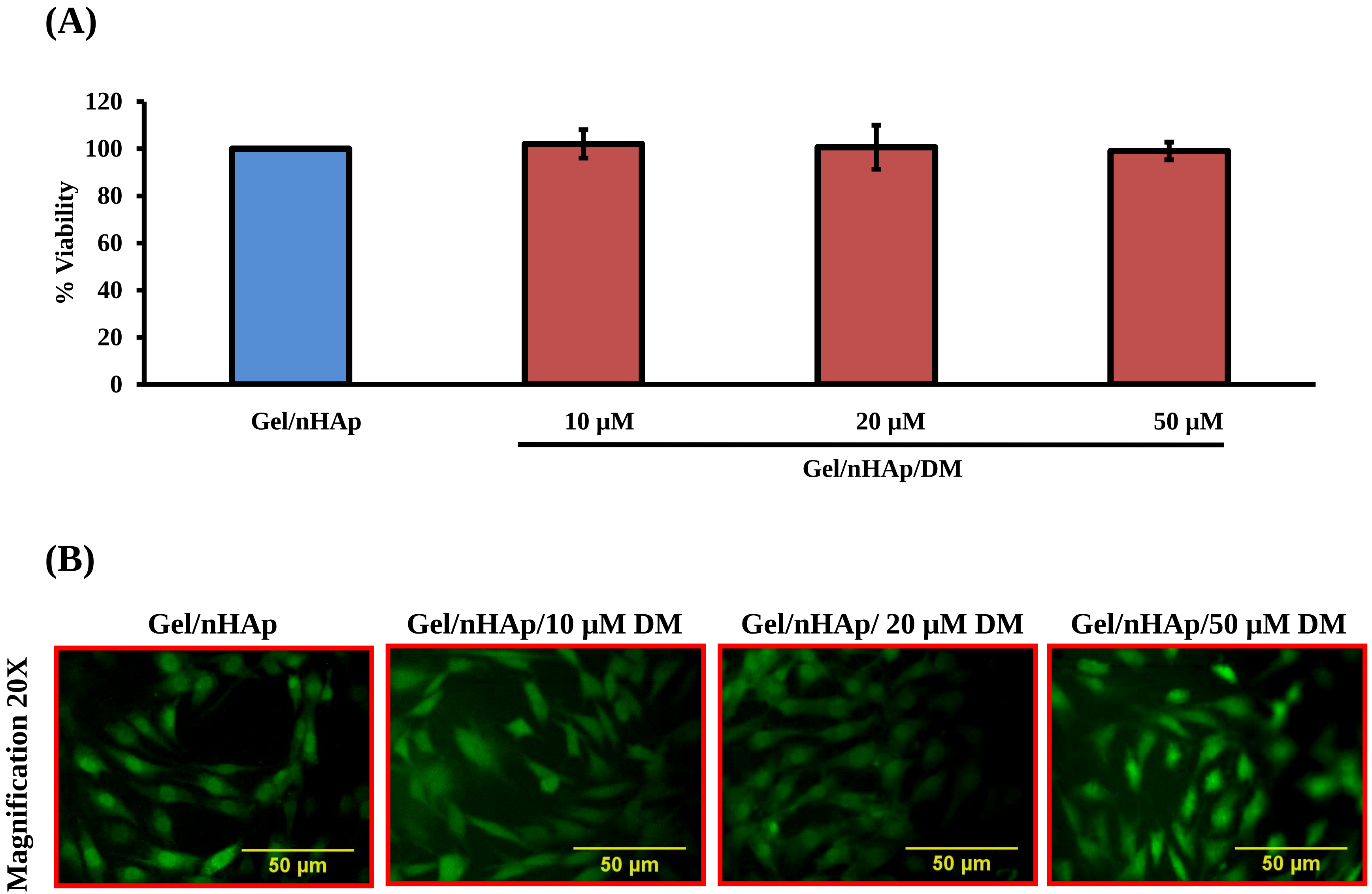

Assessment of *in vitro* cytotoxicity of scaffold. C3H10T1/2 cells were cultured on scaffolds for 72 h followed by **(A)** MTT assay and **(B)** FDA staining.

S. Figure 5

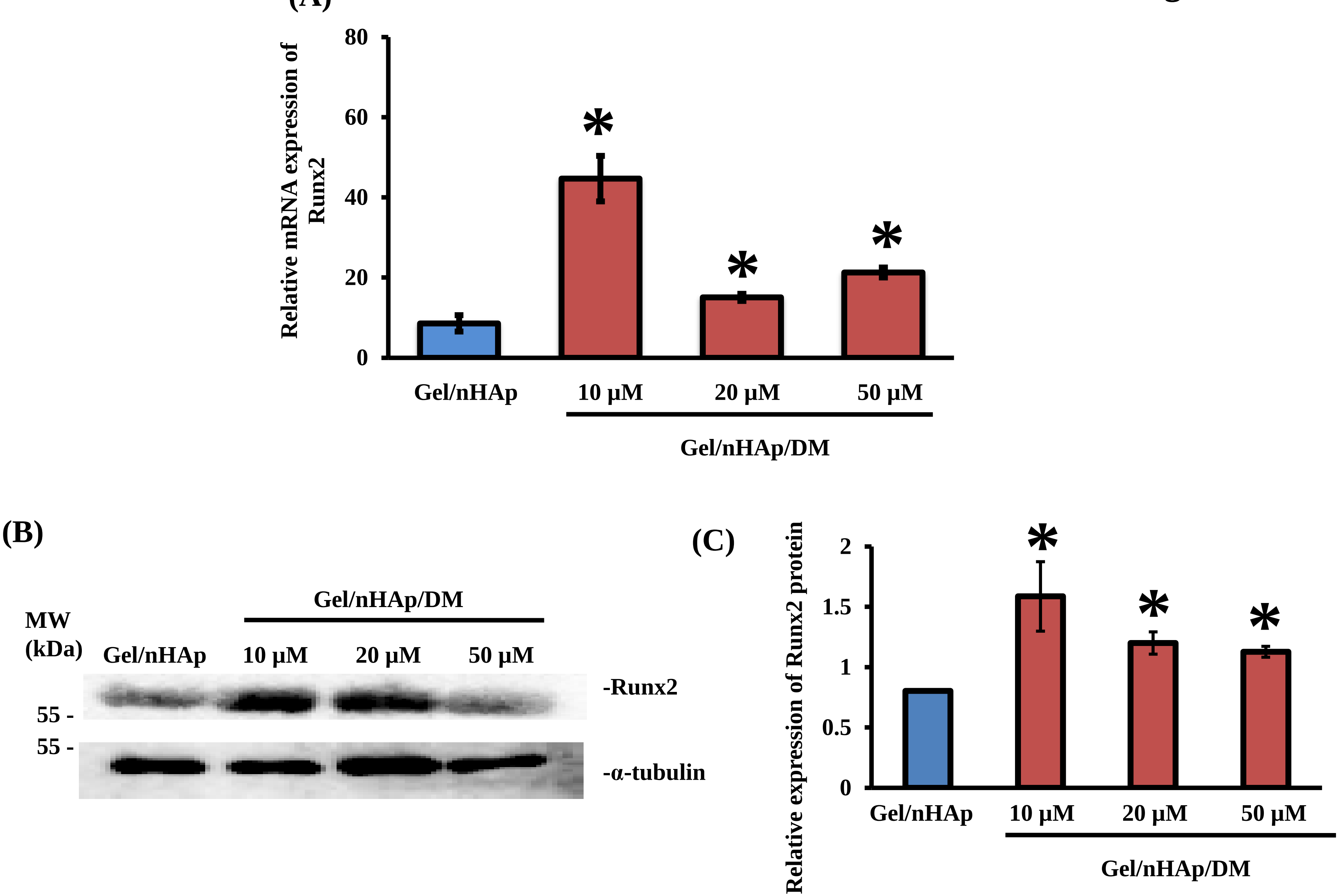

(A)
Relative mRNA expression of Runx2
80
60
40
20
0
*
Gel/nHAp
10 μM
20 μM
50 μM
Gel/nHAp/DM
(B)
Gel/nHAp/DM
MW (kDa)
Gel/nHAp
10 μM
20 μM
50 μM
55 -
55 -
-Runx2
-α-tubulin
(C)
Relative expression of Runx2 protein
2
1.5
1
0.5
0
Gel/nHAp
10 μM
20 μM
50 μM
Gel/nHAp/DM

S. Figure 5

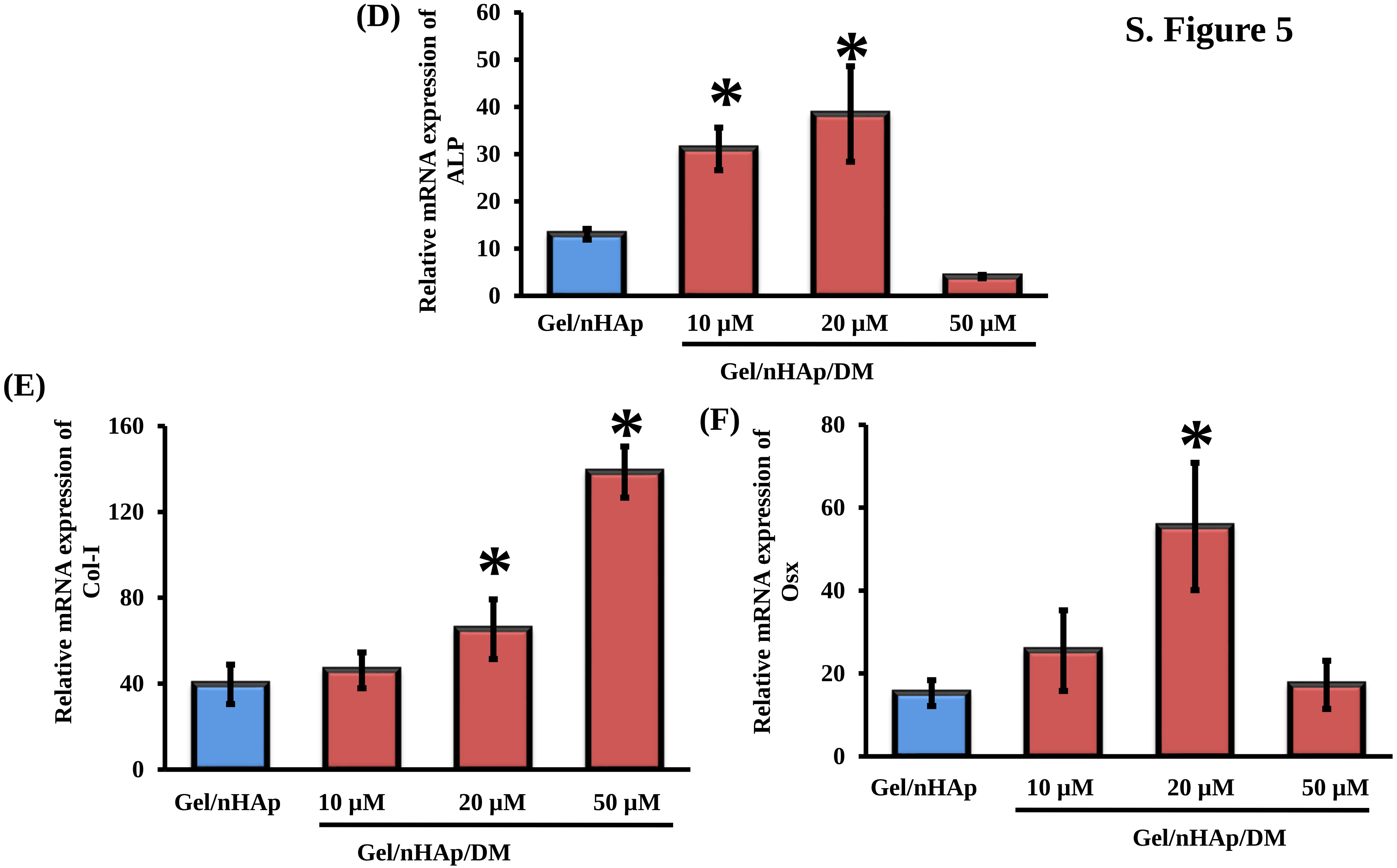

Effect of DM in osteoblast differentiation at molecular level. C3H10T1/2 were seeded on to scaffold and cultured for 14 days and RT-qPCR and western blot analyses were done. **(A)** represents Runx2 mRNA expression and **(B, C)** represent protein expression and quantification. **(D-F)** represent the relative mRNA expression of ALP, Col-I and Osx, respectively. *indicates significant increase compared to Gel/nHAp (*$p<0.05$*).

S. Figure 6

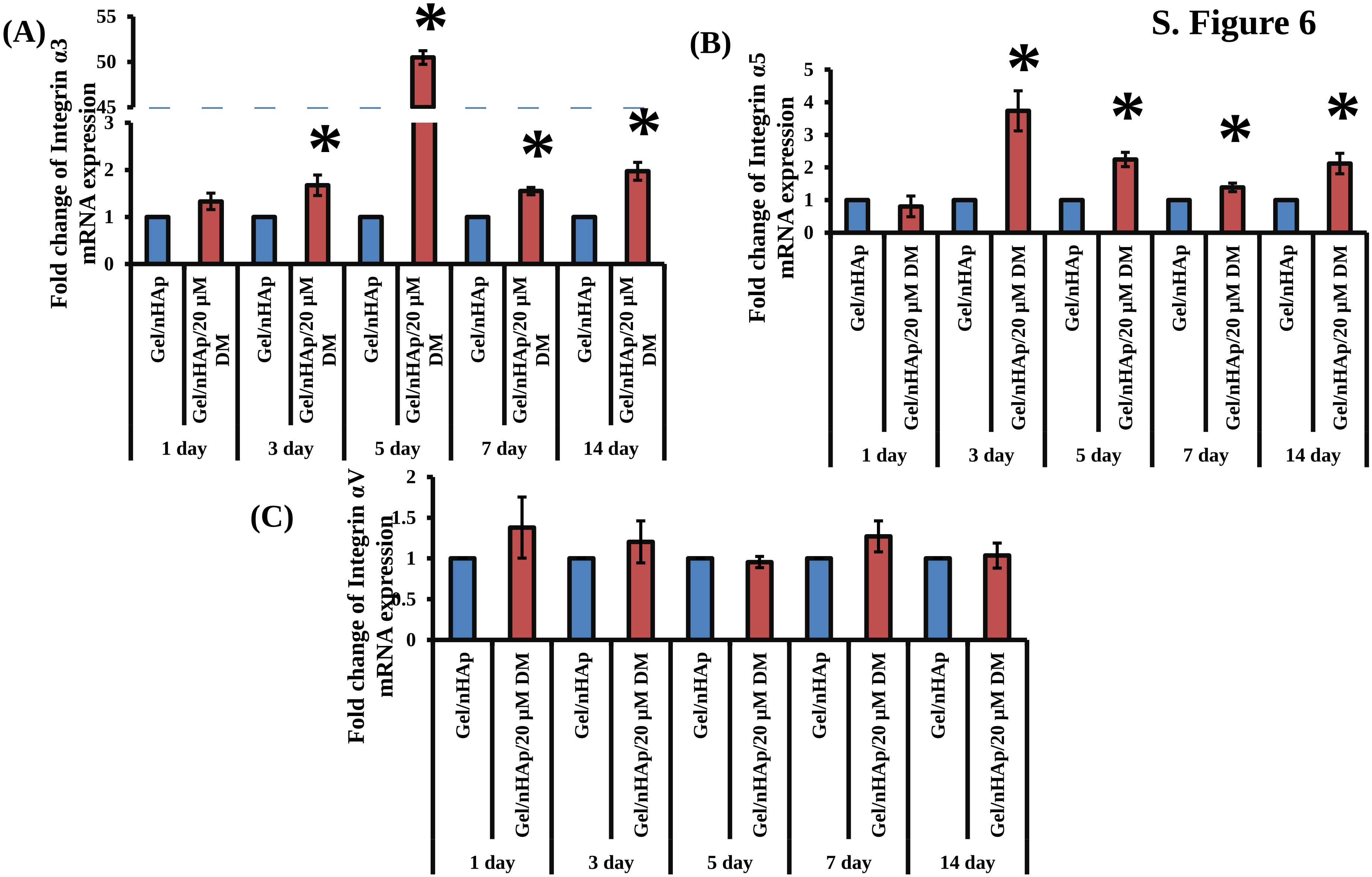

Effect of DM in cell adhesion-mediated osteogenesis. C3H10T1/2 cells were seeded on to scaffold and cultured for 1, 3, 5, 7 and 14 days followed by RNA isolation, cDNA synthesis and qPCR analysis. **(A-C)** represent the mRNA expression of Integrin α3, α5 and αV respectively. * indicates significant increase compared to respective Gel/nHAp ($p<0.05$).

S. Figure 7

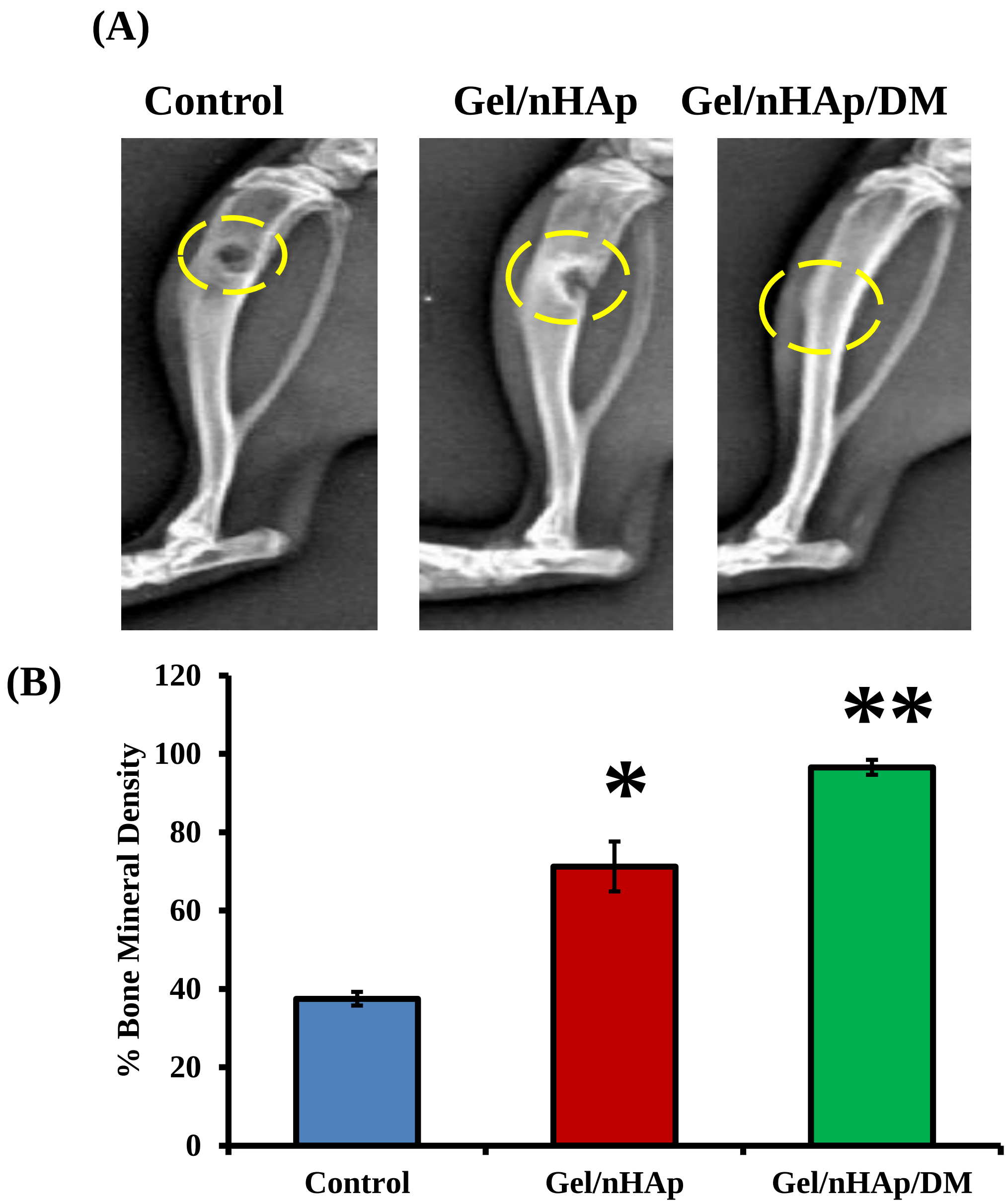

Assessment of bone formation *in vivo*. Rat tibial bone defect with 3 mm diameter was made with dental burr and post implantation evaluation was done after 4 weeks. **(A)** x-ray images and **(B)** percentage BMD of rat tibial defect of control, Gel/nHAp and Gel/nHAp/DM-treated groups. *indicates significant increase compared to control ($p<0.05$) and ** represents significant increase compared to Gel/nHAp ($p<0.05$).

S. Figure 8

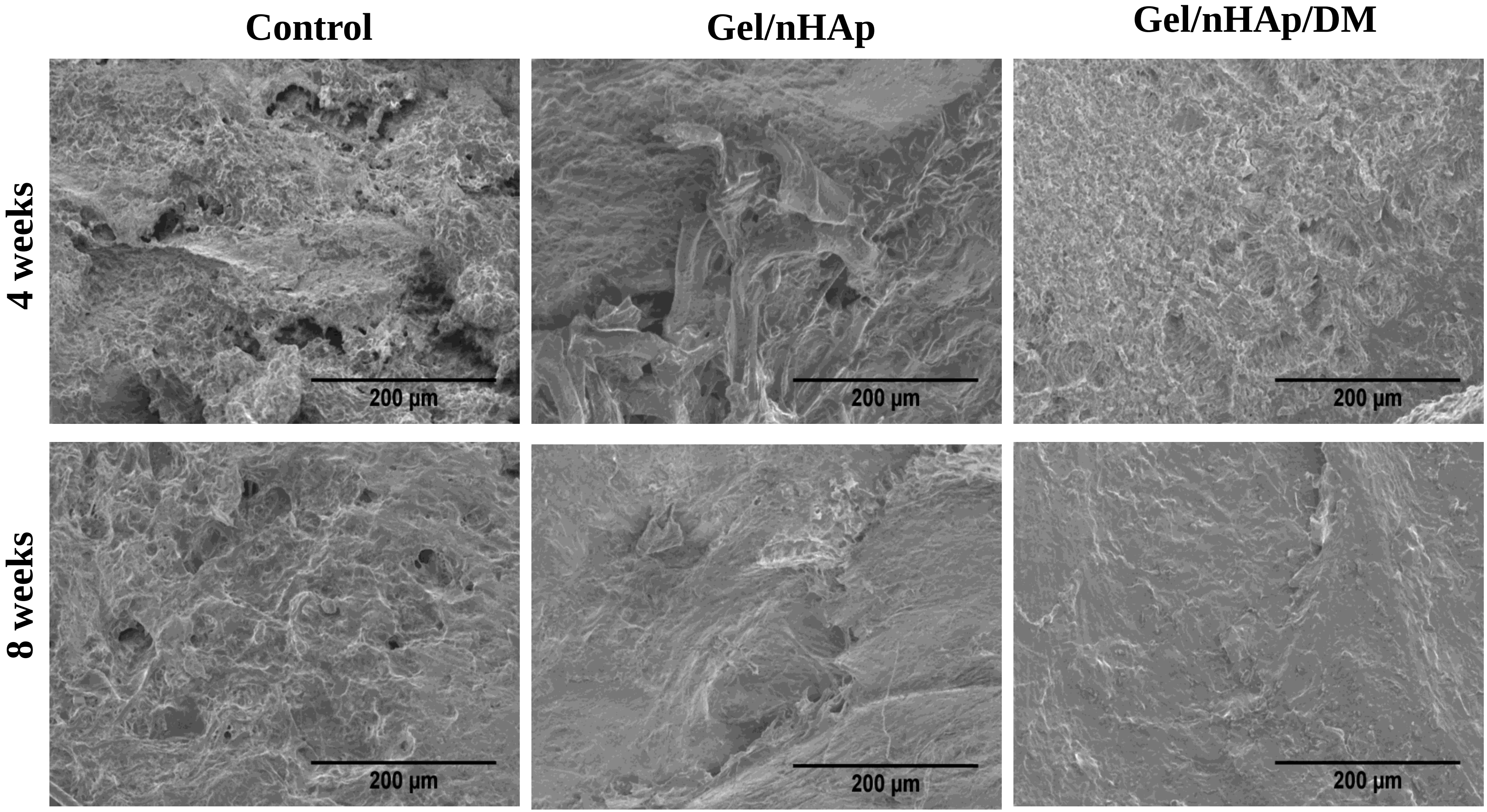

Surface morphology of newly formed bone. SEM images of bone tibial defect in control, Gel/nHAp, Gel/nHAp/DM-treated groups after 4 weeks and 8 weeks post implantation.

S. Figure 9

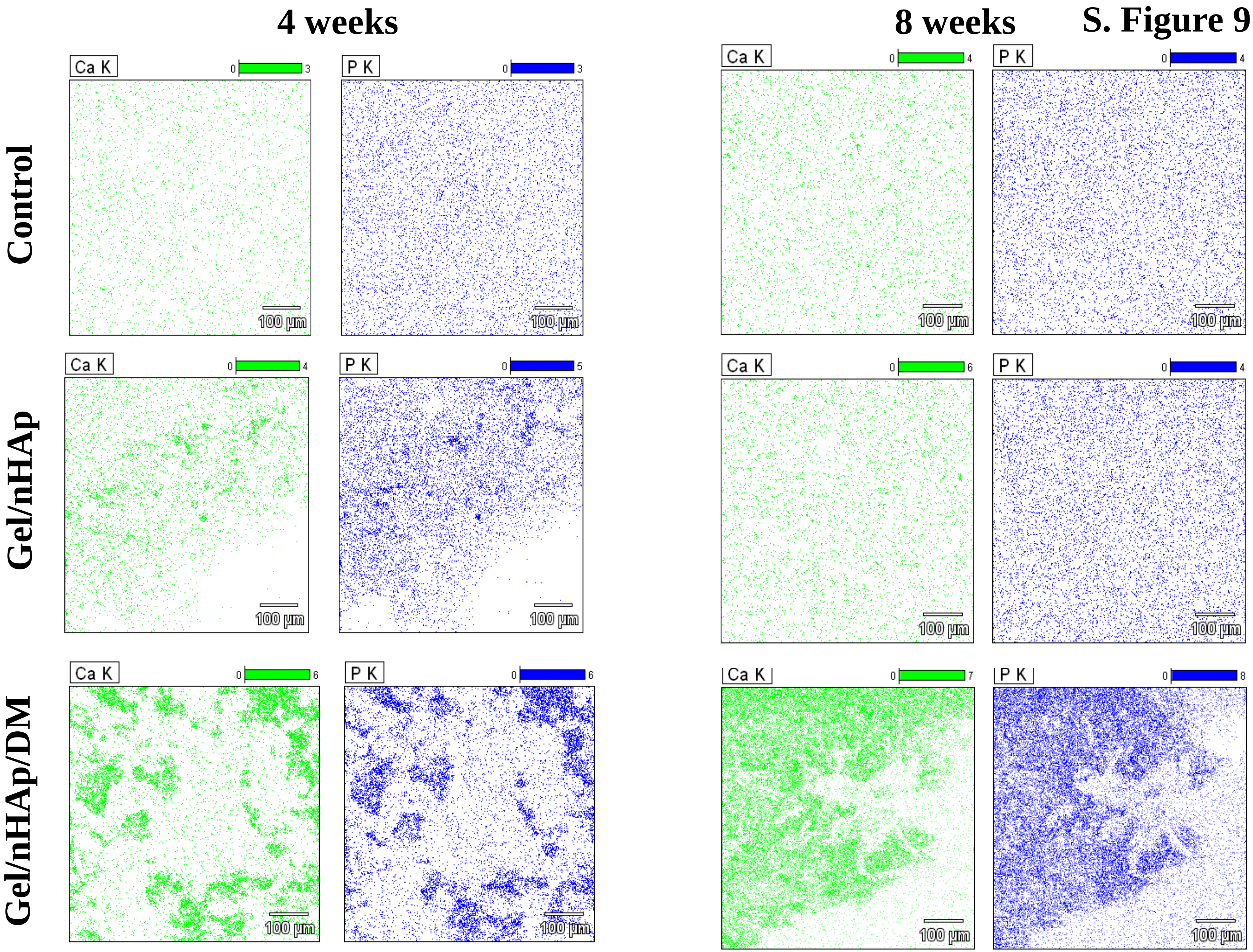

Mineralisation of newly formed bone. EDS-Mapping demonstrating distribution of calcium and phosphate on bone tibial defect in control, Gel/nHAp, Gel/nHAp/DM-treated groups after 4 weeks and 8 weeks post implantation.

S. Figure 10

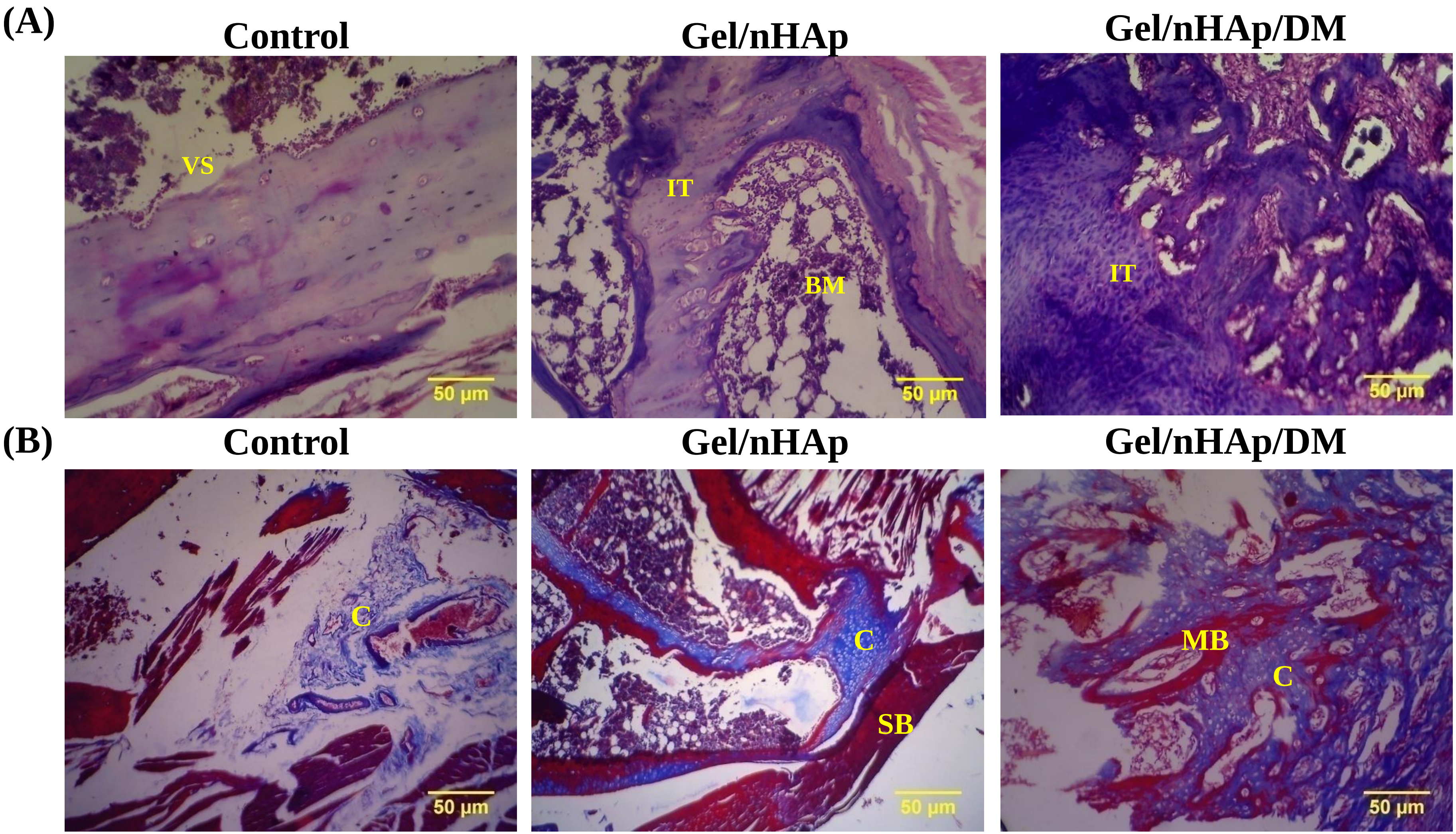

Histopathological assessment of bone formation **(A)** H and E staining and **(B)** MTS staining of decalcified bone sections of tibial defect region of control, Gel/nHAp, Gel/nHAp/DM-treated groups after 4 weeks post implantation. Denotations: VS – Vacant Space, IT- Immature Trabeculae, BM – Bone Marrow space, NB – New Bone, C – Collagen, SB – Surrounding Bone, MB – Mature Bone.

Figure 5B
(full-length blot)

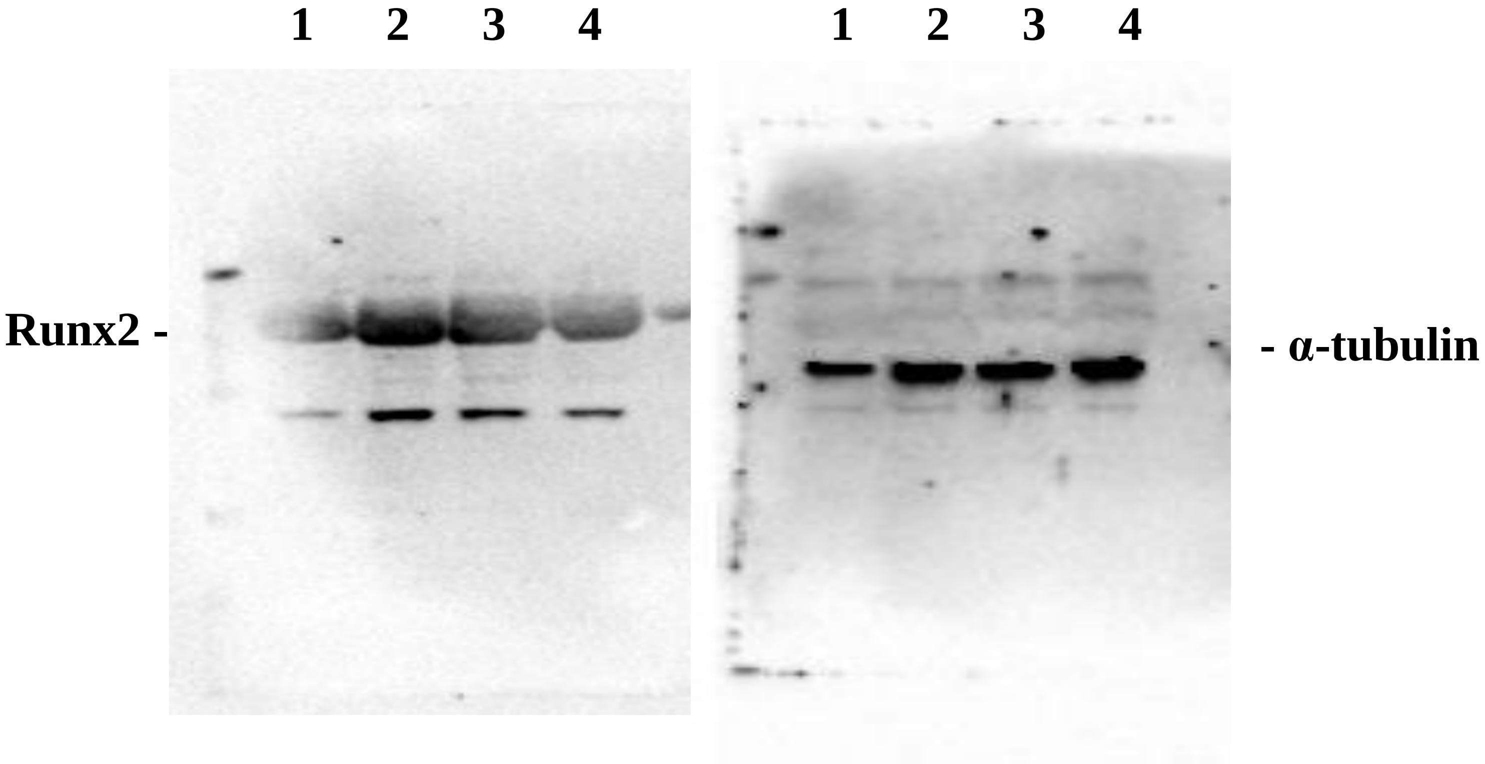

Lane 1 : Gel/nHAp
Lane 2 : Gel/nHAp/10 μM DM
Lane 3 : Gel/nHAp/20 μM DM
Lane 4 : Gel/nHAp/50 μM DM

S. Figure 5B
(full-length blot)

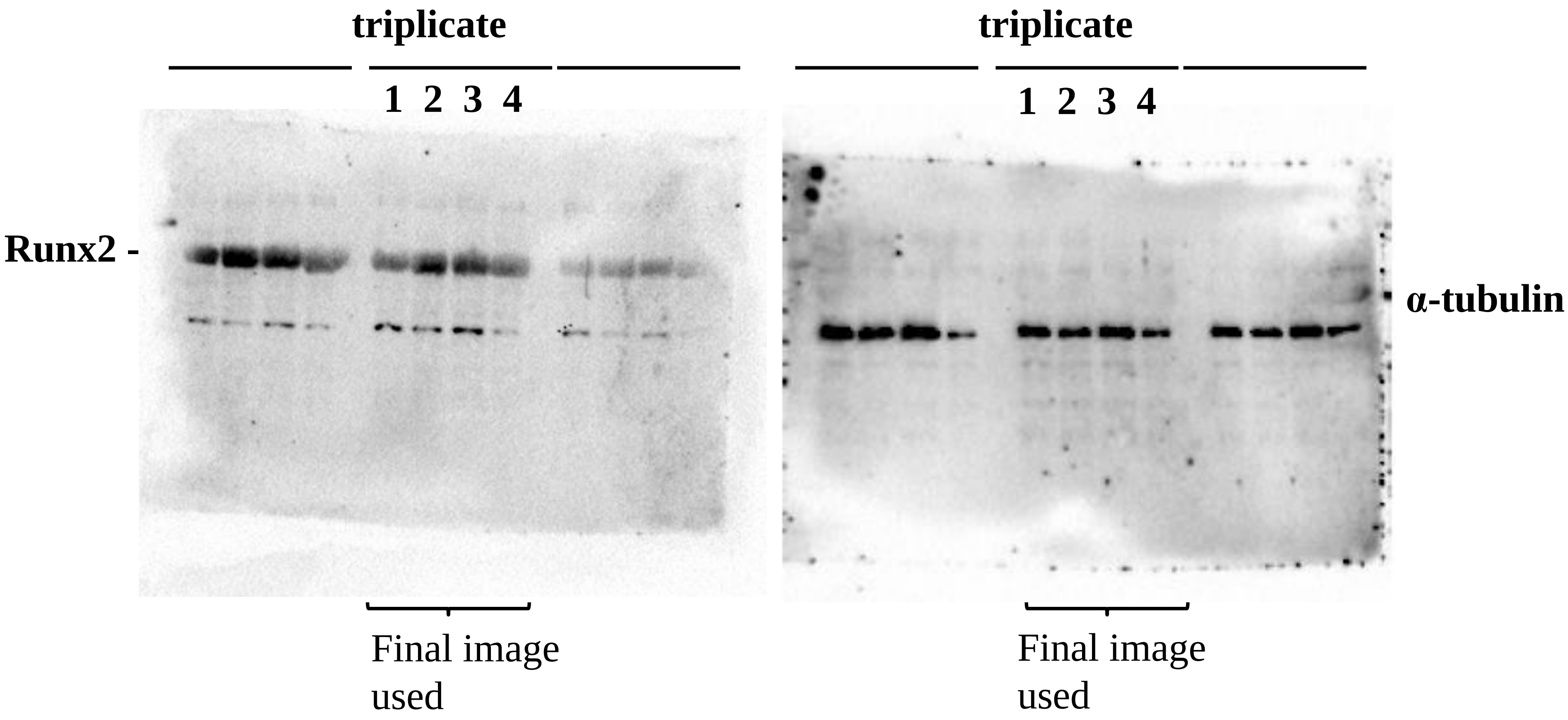

Lane 1 : Gel/nHAp
Lane 2 : Gel/nHAp/10 μM DM
Lane 3 : Gel/nHAp/20 μM DM
Lane 4 : Gel/nHAp/50 μM DM

Figure 6C
(full-length blot)

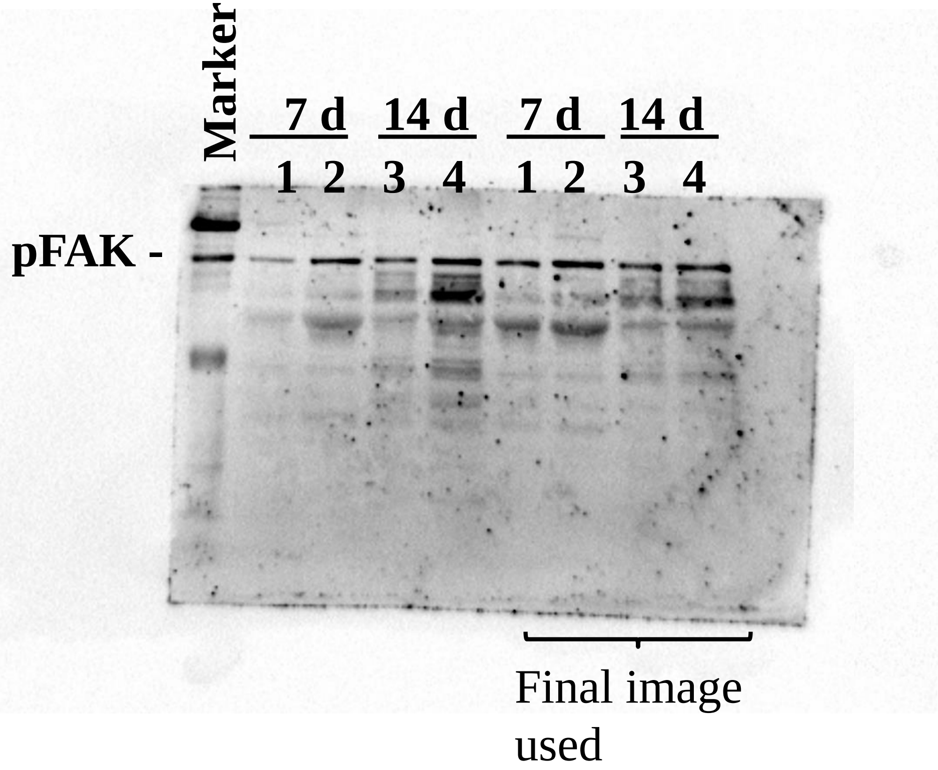

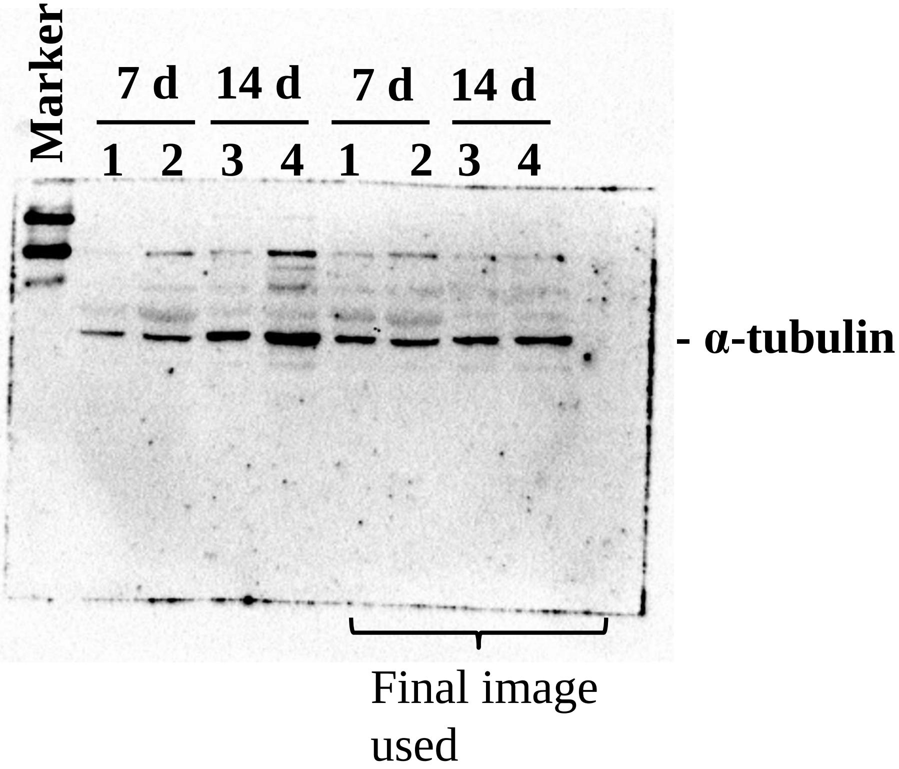

Lane 1 : Gel/nHAp
Lane 2 : Gel/nHAp/20 μM DM
Lane 3 : Gel/nHAp
Lane 4 : Gel/nHAp/20 μM DM

Figure 6C
(full-length blot)

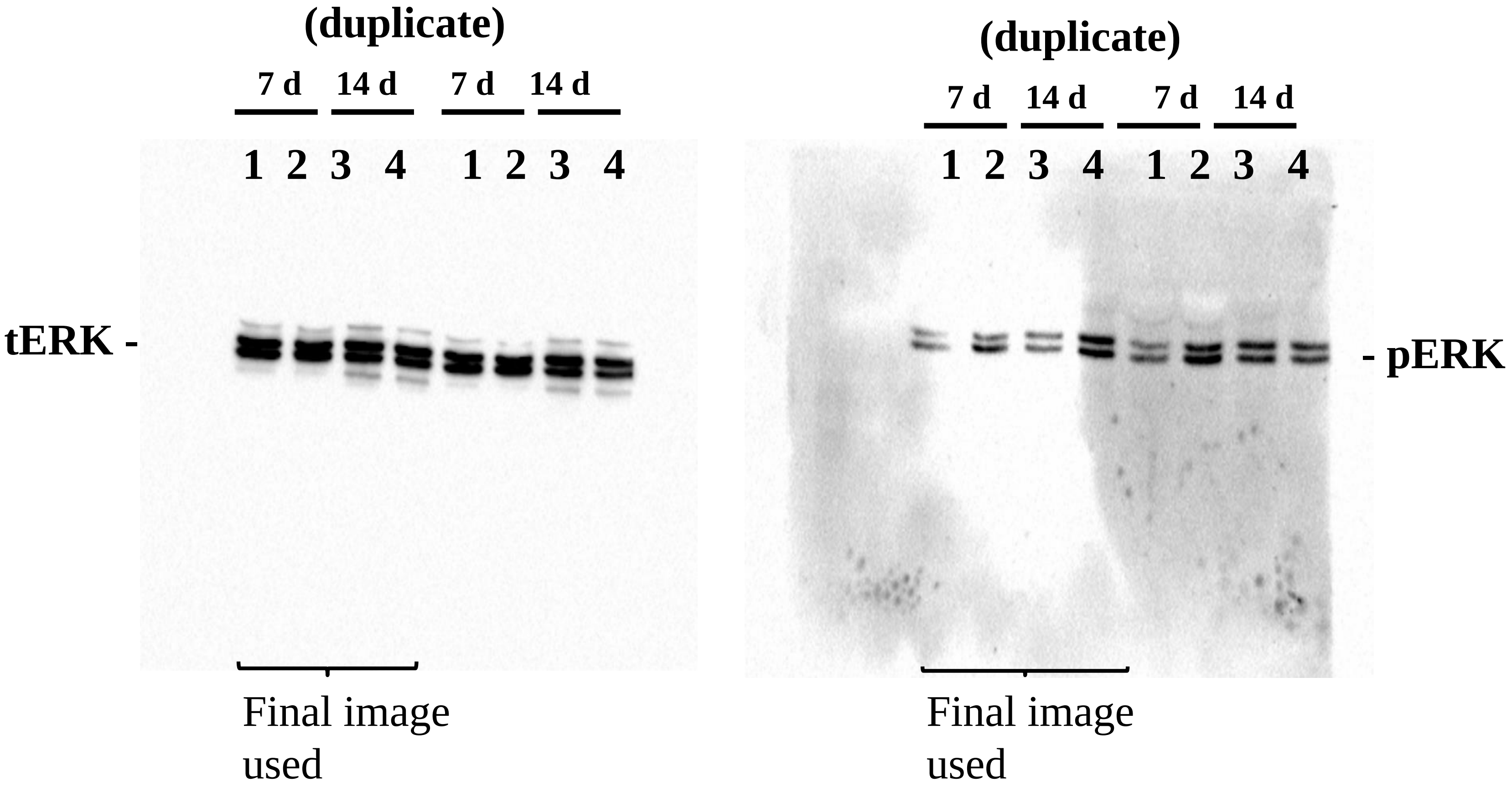

Lane 1 : Gel/nHAp
Lane 2 : Gel/nHAp/20 μM DM
Lane 3 : Gel/nHAp
Lane 4 : Gel/nHAp/20 μM DM
